# NRF2 activation in the heart induces glucose metabolic reprogramming and reduces cardiac dysfunction via upregulation of the pentose phosphate pathway

**DOI:** 10.1093/cvr/cvae250

**Published:** 2024-12-06

**Authors:** Anna Zoccarato, Ioannis Smyrnias, Christina M Reumiller, Anne D Hafstad, Mei Chong, Daniel A Richards, Celio X C Santos, Asjad Visnagri, Sharwari Verma, Daniel I Bromage, Min Zhang, Xiaohong Zhang, Greta Sawyer, Richard Thompson, Ajay M Shah

**Affiliations:** School of Cardiovascular and Metabolic Medicine and Sciences, King's College London British Heart Foundation Centre of Excellence, James Black Centre,125 Coldharbour Lane, SE5 9NU London, UK; School of Cardiovascular and Metabolic Medicine and Sciences, King's College London British Heart Foundation Centre of Excellence, James Black Centre,125 Coldharbour Lane, SE5 9NU London, UK; Comparative Biomedical Sciences, University of Surrey, Daphne Jackson Road, Manor Park Campus, GU2 7AL Guildford, UK; School of Cardiovascular and Metabolic Medicine and Sciences, King's College London British Heart Foundation Centre of Excellence, James Black Centre,125 Coldharbour Lane, SE5 9NU London, UK; School of Cardiovascular and Metabolic Medicine and Sciences, King's College London British Heart Foundation Centre of Excellence, James Black Centre,125 Coldharbour Lane, SE5 9NU London, UK; Cardiovascular Research Group, Department of Medical Biology, Faculty of Health Sciences, UiT, The Arctic University of Norway, PO box 6050 Stakkevollan, N-9037 Tromsø, Norway; School of Cardiovascular and Metabolic Medicine and Sciences, King's College London British Heart Foundation Centre of Excellence, James Black Centre,125 Coldharbour Lane, SE5 9NU London, UK; School of Cardiovascular and Metabolic Medicine and Sciences, King's College London British Heart Foundation Centre of Excellence, James Black Centre,125 Coldharbour Lane, SE5 9NU London, UK; School of Cardiovascular and Metabolic Medicine and Sciences, King's College London British Heart Foundation Centre of Excellence, James Black Centre,125 Coldharbour Lane, SE5 9NU London, UK; School of Cardiovascular and Metabolic Medicine and Sciences, King's College London British Heart Foundation Centre of Excellence, James Black Centre,125 Coldharbour Lane, SE5 9NU London, UK; School of Cardiovascular and Metabolic Medicine and Sciences, King's College London British Heart Foundation Centre of Excellence, James Black Centre,125 Coldharbour Lane, SE5 9NU London, UK; School of Cardiovascular and Metabolic Medicine and Sciences, King's College London British Heart Foundation Centre of Excellence, James Black Centre,125 Coldharbour Lane, SE5 9NU London, UK; School of Cardiovascular and Metabolic Medicine and Sciences, King's College London British Heart Foundation Centre of Excellence, James Black Centre,125 Coldharbour Lane, SE5 9NU London, UK; School of Cardiovascular and Metabolic Medicine and Sciences, King's College London British Heart Foundation Centre of Excellence, James Black Centre,125 Coldharbour Lane, SE5 9NU London, UK; School of Cardiovascular and Metabolic Medicine and Sciences, King's College London British Heart Foundation Centre of Excellence, James Black Centre,125 Coldharbour Lane, SE5 9NU London, UK; School of Cardiovascular and Metabolic Medicine and Sciences, King's College London British Heart Foundation Centre of Excellence, James Black Centre,125 Coldharbour Lane, SE5 9NU London, UK; School of Cardiovascular and Metabolic Medicine and Sciences, King's College London British Heart Foundation Centre of Excellence, James Black Centre,125 Coldharbour Lane, SE5 9NU London, UK

**Keywords:** Nuclear factor erythroid–derived 2-like 2 (NRF2), Glucose metabolism, Metabolomics, Cardiac dysfunction, Pentose phosphate pathway

## Abstract

**Aims:**

The transcription factor nuclear factor erythroid–derived 2-like 2 (NRF2) is well recognized as a master regulator of antioxidant responses and cytoprotective genes. Previous studies showed that NRF2 enhances the resistance of mouse hearts to chronic haemodynamic overload, at least in part by reducing oxidative stress. Evidence from other tissues suggests that NRF2 may modulate glucose intermediary metabolism but whether NRF2 has such effects in the heart is unclear. Here, we investigate the role of NRF2 in regulating glucose intermediary metabolism and cardiac function during disease stress.

**Methods and results:**

Cardiomyocyte-specific Keap1 knockout (csKeap1KO) mice, deficient in the endogenous inhibitor of NRF2, were used as a novel model of constitutively active NRF2 signalling. Targeted metabolomics and isotopomer analysis were employed in studies with ^13^C_6_-glucose in csKeap1KO and wild-type mice. Pharmacological and genetic approaches were utilized in neonatal rat ventricular myocytes (NRVMs) to explore molecular mechanisms. We found that cardiac-specific activation of NRF2 redirected glucose metabolism towards the pentose phosphate pathway (PPP), a branch pathway of glycolysis, and mitigated pressure overload-induced cardiomyocyte death and cardiac dysfunction. Activation of NRF2 also protected against myocardial infarction-induced DNA damage in remote myocardium and cardiac dysfunction. *In vitro*, knockdown of Keap1 upregulated PPP enzymes and reduced cell death in NRVM subjected to chronic neurohumoral stimulation. These pro-survival effects were abolished by pharmacological inhibition of the PPP or silencing of the PPP rate-limiting enzyme glucose-6-phosphate dehydrogenase. Knockdown of NRF2 in NRVM increased stress-induced DNA damage, which was rescued by supplementing the cells with either nicotinamide adenine dinucleotide phosphate (NADPH) or nucleosides, the two main products of the PPP.

**Conclusion:**

These results indicate that NRF2 regulates cardiac metabolic reprogramming by stimulating the diversion of glucose into the PPP, thereby generating NADPH and providing nucleotides to prevent stress-induced DNA damage and cardiac dysfunction.

## Graphical Abstract

**Graphical Abstract:**
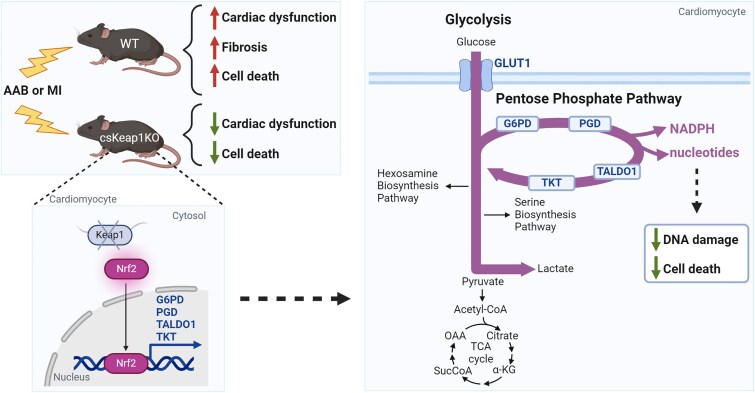

**This manuscript was handled by Consulting Editor Francesco Cosentino.Time of primary review: 43 daysSee the editorial comment for this article ‘Redirecting glucose into anabolic pathways participates in the protective effects of NRF2 activation in the heart under stress’, by T. Eschenhagen, https://doi.org/10.1093/cvr/cvaf003.**

## 1. Introduction

Heart failure (HF) is a leading cause of death worldwide and represents a substantial socioeconomic burden.^1^ In response to chronic pathological stresses, such as haemodynamic overload or myocardial infarction (MI), the heart usually develops contractile dysfunction which in the long term may progress to HF. The cardiac response to such pathological stresses is characterized by the activation of multiple pathways that collectively drive structural and functional changes, including contractile dysfunction, metabolic alterations, cardiomyocyte death, fibrosis, and ventricular dilatation.^2,3^ An increase in oxidative stress, resulting from an increase in reactive oxygen species (ROS) production and a decrease in cellular antioxidant capacity, is an important contributor to several of the processes that drive the cardiac response to pathological stresses.^4,5^

Previous studies showed that the transcription factor nuclear factor erythroid–derived 2-like 2 (NRF2) is involved in the response to pathological cardiac stress. NRF2 functions as a key controller of the cellular redox status. Under physiological conditions, NRF2 levels are kept low by binding to the inhibitor protein, Kelch-like ECH–associated protein 1 (Keap1), which promotes NRF2 ubiquitination and proteasomal degradation. During cell stress, key functional Keap1 cysteines become oxidized, thus weakening its binding to NRF2 and promoting NRF2 nuclear translocation and transcriptional activation of its target genes.^6^ These include numerous antioxidant and detoxification enzymes, such as glutathione *S*-transferases (GSTs), NAD(P)H:quinone oxidoreductase-1 (NQO1), the glutamate–cysteine ligase catalytic (GCLC), and modifier (GCLM) subunits which catalyse the rate-limiting step in glutathione (GSH) biosynthesis, glutathione reductase (GSR) which reduces oxidized glutathione, and thioredoxin reductase-1 which reduces oxidized protein thiols.^7^ Our group and others have reported that NRF2 enhances the resistance to cardiac injury and contractile dysfunction in response to pathological stresses, at least in part via activation of antioxidant pathways.^8–12^ Aside from its role as a redox homeostasis regulator, NRF2 has recently been described to induce cellular metabolic reprogramming in stress conditions.^13^ Evidence from the cancer field describes that NRF2, which in certain cancer types is associated with a malignant phenotype and cancer growth, redirects glucose and glutamine into anabolic pathways, such as the pentose phosphate pathway (PPP) and purine biosynthesis to sustain cancer cell proliferation and survival.^14^ The regulation of PPP enzymes, including the nicotinamide adenine dinucleotide phosphate (NADPH)-generating glucose-6-phosphate dehydrogenase (G6PD) and 6-phosphogluconate dehydrogenase (6-PGD), and enzymes of the non-oxidative arm of PPP, transaldolase (TALDO1) and transketolase (TKT), by NRF2 has been reported in cancer cells^13,14^ and other tissues, including inflammatory cells,^15^ liver,^16^ primary cortical astrocytes,^17^ and stem cells.^18^

In the heart, changes in energy substrate utilization and metabolism are key features of stress-induced cardiac hypertrophic remodelling.^19–21^ Interesting parallels have recently been drawn between cancer cells and failing hearts in terms of metabolic reprogramming, which may allow for increased fluxes into intermediary metabolic pathways that help cells cope with the augmented energy and biosynthetic requirements.^22^ In fact, chronic haemodynamic stress in the heart is associated with decreased fatty acid oxidation and increased glucose utilization.^19,20^ Some recent studies have explored the role of glycolytic branch pathways in settings of pathological cardiac stress although with contrasting results.^21,23^ However, whether NRF2 activation regulates glucose metabolic reprogramming in the heart under pathological stress conditions has not yet been explored. In this study, we investigate the role of NRF2 in regulating cardiac metabolism and describe the effect of constitutive activation of NRF2 on cardiac glucose metabolism using state-of-the-art metabolomics. We show that the activation of NRF2 increase glycolysis and glucose flux into the PPP, as well as the expression levels of key PPP-regulating enzymes. Activation of NRF2 protects the heart against cardiomyocyte death and cardiac dysfunction upon chronic pressure-overload or after MI, at least in part via PPP-mediated reduction of cardiomyocyte DNA damage.

## 2. Methods

### 2.1 Mouse models

All animal procedures were carried out under the authority of a Home Office Project Licence in accordance with the Guidance on the Operation of the Animals (Scientific Procedures) Act, 1986 (UK). All procedures conformed to the guidelines from Directive 2010/63/EU of the European Parliament on the protection of animals used for scientific purposes. Cardiomyocyte-targeted knockout of Keap1 was achieved by crossing Keap1 F/F mice^24^ with cardiomyocyte-specific Cre (αMHC-Cre) mice, all on a C57BL/6N background. Aortic constriction was performed on male csKeap1KO mice and wild-type (WT) littermates (16–18 g) by a single surgeon, using suprarenal banding with a 27-G constriction under 2% isoflurane/98% oxygen anaesthesia.^25^ Sham constriction involved identical surgery apart from band placement. Animals were studied up to 6 weeks post-surgery. Female csKeap1KO mice and WT littermates ∼12 weeks of age underwent left anterior descending (LAD) coronary artery ligation under 2% isoflurane/98% oxygen anaesthesia.^26^ Sham groups underwent identical surgery except for ligation. Experimental analyses were done at 4 weeks after surgery. Mice were humanely killed in accordance with Schedule 1 of the Animals (Scientific Procedures) Act 1986 and the guidelines from Directive 2010/63/EU by dislocation of the neck, followed by exsanguination to confirm death.

### 2.2 Echocardiography

The animals were imaged^27^ under 1.5% isoflurane using a Visualsonics Vevo 2100 ultrasound system with a 40 MHz transducer. The analysis of data was performed using the VevoStrain software package (Visualsonics, Toronto, Canada).

### 2.3 Langendorff heart perfusion

The preparation and perfusion of the hearts was performed in the Langendorff mode. In brief, mice were anaesthetized with an i.p. injection of pentobarbital sodium (90 mg/kg)-containing heparin (5000 U/kg). The hearts were excised and cannulated and subsequently perfused with Krebs–Henseleit (KH) bicarbonate buffer containing 5 mmol/L glucose, 0.2 mmol/L pyruvate, 0.5 mmol/L glutamine, 1.5 mmol/L lactate, 0.4 mmol/L Na-octanoate, 50 mU/L insulin, 118.5 mmol/L NaCl; 5.9 mmol/L KCl; 0.48 mmol/L ethylenediaminetetraacetic acid (EDTA); 1.16 mmol/L MgSO_4_; 2.2 mmol/L CaCl_2_; 25 mmol/L NaHCO_3_ (37°C, pH 7.2). After 20 min equilibration, hearts were perfused for 20 min with a modified KH buffer containing uniformly labelled glucose [5 mmol/L (U-^13^C)glucose; Cambridge Isotope Laboratories, Inc., Tewksbury, MA] instead of standard glucose, with all other components unchanged. The perfusion pressure was set to 60 mmHg and monitored using an intraventricular balloon. Immediately after perfusion, hearts were freeze-clamped for metabolic quenching and stored at −80°C.

### 2.4 Metabolite extraction from tissue samples for liquid chromatography–mass spectrometry

Frozen heart tissue (∼40–50 mg) was lyophilized overnight (−55°C) and subsequently ground in liquid nitrogen with a mortar and pestle. Pulverized tissue was then resuspended in a 4°C cold chloroform:methanol solution (2:1, v/v) and vortexed. Resuspended tissues were incubated for 1 h at 4°C, including 3 pulses of sonication (8 min each). Subsequently, samples were centrifuged (0°C, 10 min, 16 000 *g*), and the resulting supernatant was transferred to a new tube and dried at room temperature using a SpeedVac concentrator (Thermo Fisher Scientific, Hampton, NH). The pellet was re-extracted by repeating this procedure with methanol:water solution (2:1, v/v). The dried metabolite extracts were reconstituted in chloroform:methanol:water solution (1:3:3, v/v/v) and incubated for 10 min before a final centrifugation step (0°C, 5 min, 16 000*g*). After spinning, the upper polar phase was isolated and dried in a SpeedVac concentrator. Dried polar fractions were stored at −80°C until mass spectrometry (MS) analysis. Neonatal rat ventricular myocytes (NRVMs) were isolated as described below, seeded at 0.5 × 10^6^ in 6-well plates, and transduced with adenoviral vectors as indicated. Forty-eight hours later, cells were incubated with [U-^13^C]glucose (Cambridge Isotope Laboratories, Inc.; 20 mM) for 18 h in Dulbecco's modified Eagle's medium (DMEM) media (D5030; Sigma-Aldrich, Merck KGaA, Darmstadt, Germany) supplemented with 1 mM pyruvate and 4 mM glutamine. At the labelling endpoint, cells were washed with ice-cold phosphate-buffered saline (PBS) and quenched with 1:1:1 chloroform:methanol:water (v/v/v). Cells were scraped off the plate, and the cell suspension was placed in a tube shaker for 20 min at 4°C, followed by centrifugation at 16 000 *g* at 4°C for phase separation. The polar phase was collected and dried using a SpeedVac (Thermo Fisher Scientific). The dried polar fractions were stored at −80°C until MS analysis.

### 2.5 Liquid chromatography–MS analysis

The liquid chromatography (LC)–MS method has been previously described.^28^ Briefly, the dried polar metabolite fractions were reconstituted in acetonitrile/water (v/v 3/2), vortexed and centrifuged at 16 000 *g* for 3 min before analysis on a 1290 Infinity II ultrahigh-performance liquid chromatography (HPLC) system coupled to a 6546-quadrupole time-of-flight mass spectrometer (Agilent Technologies, Santa Clara, CA). Samples were separated on a Poroshell 120 HILIC-Z column (100 × 2.1 mm, 2.7 μm; Agilent Technologies) attached to a guard column (5 × 2.1 mm, 2.7 µm) and analysed in negative ionization mode using water with 10 mmol/L ammonium acetate (Solvent A) and acetonitrile with 10 mmol/L ammonium acetate (Solvent B), both solvents containing 5 µmol/L Infinity Lab deactivator additive (Agilent Technologies). The elution gradient used was as follows: isocratic step at 95% B for 2 min, 95–65% B in 12 min, maintained at 65% B for 3 min, then returned to initial conditions over 1 min, and then the column was equilibrated at initial conditions for 8 min. The flow rate was 0.25 mL/min; the injection volume was 1 μL, and the column oven was maintained at 30°C. Feature annotation and metabolite identification were based on accurate mass and standard retention times with a tolerance of ±5 ppm and ±0.5 min, respectively, and performed with MassHunter Profinder (version 10.0.2; Agilent Technologies) using our in-house curated metabolite library (see Supplementary material online, *Table S1*) based on metabolite standards (Sigma-Aldrich). ^13^C label incorporation levels were normalized to the natural occurrence of ^13^C isotopes and are represented as corrected abundance percentages. Samples were run in one batch and injected into technical duplicates. Downstream pathway enrichment analysis was performed using MetaboAnalyst 5.0 Software.^29^

### 2.6 ROS assessment using HPLC detection of dihydroethidium oxidation products

ROS levels were directly assessed through the detection of the dihydroethidium (DHE) oxidation products, 2-hydroxyethidine and ethidium (E), readouts of superoxide and of H_2_O_2_ and other ROS, respectively. Briefly, the hearts were harvested and ground in liquid nitrogen with a pestle and mortar. Pulverized tissue was weighed (∼20 mg) and incubated with 0.1 mmol/L DHE [PBS containing 0.1 mmol/L diethylenetriamine pentaacetic acid (DTPA)] at 37°C for 30 min. The buffer was removed, and the sample washed with PBS–DTPA. DHE and oxidized products were extracted with acetonitrile (0.5 mL) and sonicated (3 × 30 s, 8 W). Samples were spun down (18 000 *g*, 10 min, 4°C), the supernatants were collected and dried using a SpeedVac (Thermo Fisher Scientific). The samples were stored at −80°C, and then dissolved in 0.120 mL PBS–DTPA prior to injecting into an HPLC system and analysing, as previously described.^30,31^

### 2.7 Histology

Hearts were arrested in diastole with 5% KCl and fixed with 2% paraformaldehyde either for 6 h at room temperature or overnight at 4°C. Subsequently, 6 µm paraffin-embedded transverse cross-sections were stained with fluorescein isothiocyanate–conjugated wheat germ agglutinin (FITC-WGA, Vector RL-1022) to outline cardiomyocytes. Interstitial fibrosis was assessed by blinded quantitative image analysis (Volocity; PerkinElmer, Waltham, MA) of Picrosirius red-stained sections.^10^ Apoptosis was assessed by terminal deoxynucleotidyl transferase dUTP nick-end labelling (TUNEL) staining following the manufacturer’s instructions (Millipore S7110 kit). Infarct size after LAD ligation was measured by Evans blue dye (1%) staining, as previously described.^32^ To quantify DNA damage, sections were stained with an anti-γH2A.X antibody [Phospho-Histone H2A.X (Ser139) (20E3); Cell Signaling, Danvers, MA, #9718]. Imaging was done on a Nikon spinning disc confocal microscope.

### 2.8 Western blotting

Heart tissue samples or pelleted cardiomyocytes were homogenized and lysed in hypotonic lysis buffer (50 mmol/L 4-(2-hydroxyethyl)piperazine-1-ethane-sulphonic acid, pH 7.4, 10 mmol/L KCl, 5 mmol/L EDTA, 5 mmol/L ethylene glycol bis(2-aminoethyl ether)-N,N,N',N'-tetraacetic acid, 2 mmol/L MgCl_2_, 2 mmol/L dithiothreitol, 0.1% NP-40, protease inhibitor cocktails and Ser/Thr and Tyr phosphatase inhibitor cocktails; Sigma-Aldrich, UK). Protein concentration for each sample was measured using bicinchoninic acid assay (Pierce, UK). Heart tissue homogenates or cell lysates were separated by sodium dodecyl sulphate–polyacrylamide gel electrophoresis and transferred onto nitrocellulose membranes. Antibodies used were the following: G6PD (Abcam, Cambridge, UK; ab210702), 6-PGD (Abcam, ab129199), transaldolase 1 (Thermo Fisher Scientific; A304–326A-T), TKT (Cell Signaling, #8616), Nrf2 (Abcam, ab137550), Keap1 (Santa Cruz Biotechnology, Dallas, TX, sc-15246), tubulin (Abcam, ab7291), GST A2, GSTA2 (Sigma, SAB1401163), p62 (Cell Signaling, #39749). Protein band quantification was undertaken using an Odyssey Li-Cor imaging system (Li-Cor Biosciences, Lincoln, NE).

### 2.9 cDNA synthesis and real-time quantitative polymerase chain reaction

Total RNA was isolated from heart tissues or cardiomyocyte samples according to the manufacturer’s protocol (Qiagen, Venlo, The Netherlands). cDNA was synthesized using Oligo-dTs and M-MLV RT (Promega, Madison, WI). Reverse transcriptase–quantitative polymerase chain reaction was performed with the StepOnePlus System (Applied Biosystems-Thermo Fisher Scientific) using SYBR Green and the comparative Ct method was used, with cytoskeletal β-actin levels used for normalization. Primer sequences are listed in Supplementary material online, *Table S2*.

### 2.10 Neonatal rat ventricular myocytes

Primary neonatal rat ventricular myocytes (NRVMs) were isolated from 1 to 2 days old Sprague–Dawley (SD) rats (Charles River Laboratories, Wilmington, MA) as previously described.^33^ P1–P2 SD rats were humanely killed in accordance with Schedule 1 of the Animals (Scientific Procedures) Act 1986 and the guidelines from Directive 2010/63/EU by dislocation of the neck, followed by exsanguination to confirm death. Cells were cultured in plating medium (DMEM High Glucose, MEM199, Horse serum, non-heat-inactivated foetal bovine serum, non-essential amino acids, glutamine, Pen/Strep) for 24 h before switching to a serum-free medium containing DMEM High Glucose, MEM199, Glutamine, Pen/Strep. Cells were transduced with adenoviral vectors (Welgen Inc., Worcester, MA) expressing a short hairpin sequence targeted against NRF2 (shNRF2) or a non-targeting sequence (shCtr) at MOI 500. For the knockdown of Keap1 or G6pd, short interfering RNAs (siRNAs) were purchased from Thermo Fisher Scientific (Silencer® Select: s138759; Silencer® Select: s127750), and control siRNA was obtained from Dharmacon (ON-TARGETplus Non-targeting Pool). NRVMs were transfected using TurboFect Transfection Reagent (Thermo Fisher Scientific) following the manufacturer’s instructions. NRVMs were treated for 24 or 8 h with isoproterenol 100 µmol/L (Isoprenaline hydrochloride, I5627; Sigma-Aldrich), 6-aminonicotinamide (6-AN) 2.5 mmol/L (A68203; Sigma-Aldrich), nucleosides 0.3 mmol/L [EmbryoMax® Nucleosides (100×); Merck, Darmstadt, Germany] or NADPH 0.3 mmol/L (NADPH, Tetrasodium Salt, 481973; Merck).

### 2.11 Immunofluorescence and confocal imaging

NRVMs were washed with PBS and fixed in 4% paraformaldehyde for 20 min at room temperature and permeabilized in 0.1% v/v Triton™ X-100 in PBS. Blocking and antibody incubations were performed in 2% w/v bovine serum albumin. For cell viability assays, 7-Aminoactinomycin D (7-AAD; Invitrogen, Waltham, MA, A1310) and Hoechst 33342 (Invitrogen, H3570) were used following the manufacturer’s instructions. Images were captured using a Nikon Spinning disc confocal and analysis was performed using ImageJ.

### 2.12 Statistical analysis

All data are represented as mean ± standard error of the mean (SEM). Unpaired, two-tailed Student’s *t*-test was used to compare differences between the two groups. For multiple group comparisons with one variable, one-way analysis of variance (ANOVA) was performed, followed by Tukey multiple comparisons test. For multiple group comparisons with two or more variables, two-way ANOVA was conducted, followed by Tukey multiple comparisons test. For mass isotopologue distribution analysis, one-way ANOVA followed by Bonferroni multiple comparison test was performed. The number of replicates is indicated in the figure legends. Statistical analyses were performed with GraphPad Prism 9.5.1.

## 3. Results

### 3.1 Cardiac-specific constitutive activation of NRF2 induces antioxidant responses and metabolic reprogramming in the heart

**Figure 1:**
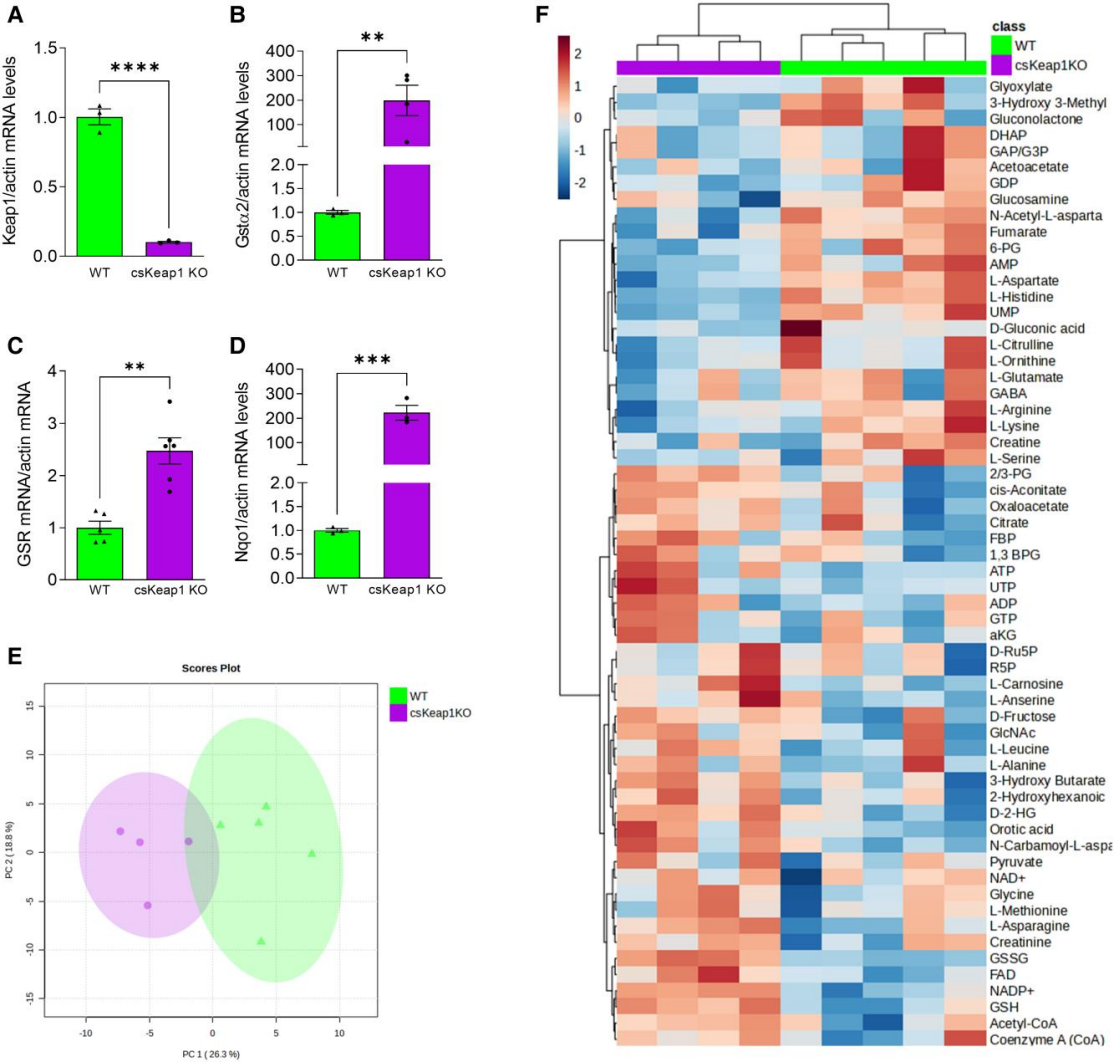
Cardiac-specific activation of NRF2 induces metabolic reprogramming in the heart. *A*) mRNA levels of Keap1 and NRF2 targets *B*) glutathione-S-transferase α2 (*Gsta2*), *C*) glutathione-disulphide reductase (*Gsr*), *D*) nicotinamide adenine dinucleotide phosphate-quinone-oxidoreductase 1 *(Nqo1)* in the hearts of csKeap1KO and WT control mice. *n* ≥ 3/group. *E*) Principal component analysis of targeted metabolomic profile of csKeap1KO vs. WT hearts, *n* ≥ 4/group. *F*) Heat map showing hierarchical clustering of metabolite abundance for the top 60 features in WT vs. csKeap1KO hearts. Data are presented as mean ± SEM. **P < 0.01, ***P < 0.001, ****P < 0.0001 and ns, not significant by unpaired Student’s *t*-test.

To study whether NRF2 mediates metabolic reprogramming in the heart, we generated a mouse model of cardiac-specific constitutive activation of NRF2 by knocking out Keap1 in cardiomyocytes. Conditional Keap1-floxed mice^24^ were crossed with cardiomyocyte-specific Cre transgenic mice [αMHC (α-myosin heavy chain)-Cre] to generate cardiomyocyte-specific Keap1 knockout (csKeap1KO) mice (see Supplementary material online, *Figure S1A*). Because the Keap1-floxed model exhibits some hypomorphism,^35^ we compared csKeap1KO mice with WT littermates. csKeap1KO mice and WT littermates manifested no obvious differences at birth, had similar patterns of growth (see Supplementary material online, *Figure S1B*), and showed comparable baseline contractile function and cardiac dimensions by echocardiography (see Supplementary material online, *Figure S1C–H*). csKeap1KO hearts had significantly lower levels of Keap1, increased levels of NRF2, and increased mRNA expression levels of NRF2 targets, such as *Gsta2*, *GSR*, *Nqo1*, and *Gclm* confirming an activation of NRF2 (*Figure 1A–D*, Supplementary material online, *Figure S2A*, *H*–*L*). csKeap1KO hearts had higher levels of glutathione (GSH and GSSG) and an increased GSH/GSSG ratio compared with WT littermates (see Supplementary material online, *Figure S2D–F*). Direct assessment of ROS levels by HPLC-based detection of DHE oxidation products revealed a significant reduction in H_2_O_2_ and other ROS in csKeap1KO compared with WT hearts but no difference in superoxide levels, in keeping with the more reduced glutathione redox state (see Supplementary material online, *Figure S2B* and *C*). The NADP^+^/NADPH ratio was increased in csKeap1KO hearts consistent with increased NADPH utilization (see Supplementary material online, *Figure S2G*). To assess whether NRF2 activation in the heart mediates metabolic reprogramming, we performed LC–MS-based targeted metabolomics encompassing glycolytic, branch pathway, and trichloroacetic acid (TCA) cycle metabolites on WT and csKeap1KO hearts. Principal component analysis, performed using MetaboAnalyst 5.0, identified a clear separation and clustering between WT and csKeap1KO heart metabolite profiles (*Figure 1E*). The majority of the top 60 metabolites that differentially clustered between the two genotypes comprised metabolites related to glutathione metabolism, glycolysis, PPP, and nucleotides (*Figure 1F*). Overall, these data indicate that activation of NRF2 in the heart alters its metabolite profile.

### 3.2 NRF2 activation in the heart increases glucose utilization and flux into the PPP

**Figure 2:**
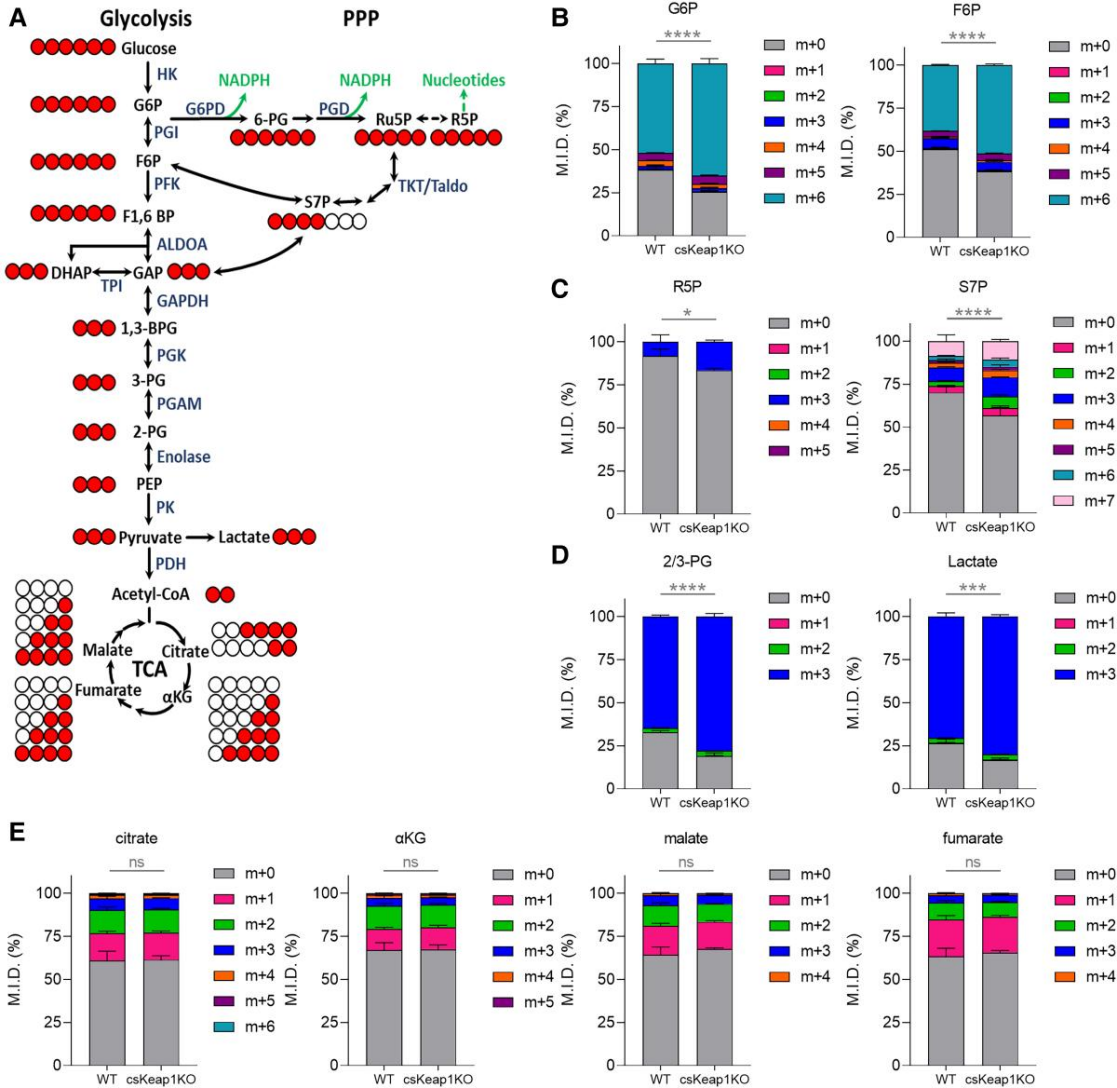
Effect of constitutive activation of NRF2 in the heart on ^13^C enrichment of metabolites after *ex vivo* Langendorff perfusion of [U-^13^C]glucose. *A*) Schematic representation of [U-^13^C]glucose carbon labelling through glycolysis, PPP, and TCA cycle (filled circles indicate ^13^C-labelled carbons, and empty circles indicate ^12^C carbons). *B*) ^13^C-glucose incorporation into upper glycolytic metabolites: G6P and F6P (mass isotopologue distribution, M.I.D.), *C*) ^13^C-glucose incorporation into PPP metabolites: R5P and S7P (M.I.D.), *D*) ^13^C-glucose incorporation into lower glycolytic metabolites: 2/3PG and lactate (M.I.D.), *E*) ^13^C-glucose incorporation into TCA cycle metabolites: citrate, alpha-ketoglutarate, malate, and fumarate (M.I.D.) in WT vs. csKeap1KO heart following *ex vivo* Langendorff perfusion of [U-^13^C]glucose, *n* = 3/group. Mass isotopologue distribution refers to the relative abundance (in percentage) of isotopically labelled molecules with different masses within a given metabolite. *m* + 0, refers to unlabelled isotopologue, *m* + 1 to isotopologues containing one ^13^C atom, *m* + 2 to isotopologues with two ^13^C atoms, etc.^34^ Data are presented as mean ± SEM. *P < 0.05, ***P < 0.001, ****P < 0.0001 and ns, not significant by one-way ANOVA followed by Bonferroni multiple comparison test for mass isotopologue comparisons.

Given the differences observed in abundance of metabolites related to glucose metabolism, we assessed whether glucose utilization and flux were altered in the csKeap1KO hearts. We undertook stable-isotope-resolved metabolomics of *ex vivo* csKeap1KO and WT Langendorff-perfused hearts using uniformly labelled glucose ([U-^13^C]glucose) followed by targeted LC–MS metabolomics and ^13^C-isotopomer analysis. A principal component analysis showed that csKeap1KO hearts presented a distinct ^13^C metabolite enrichment when compared with WT littermates (see Supplementary material online, *Figure S3A*). We measured U-^13^C-glucose incorporation through glycolysis, TCA cycle, PPP, and other glycolytic branch pathways (*Figure 2A*). Our results showed a significant increase in csKeap1KO hearts compared with WT in the percentage of ^13^C label enrichment of glucose, glucose-6-phosphate (G6P), fructose-6-phosphate (F6P; *Figure 2B*, Supplementary material online, *Figure S3B*), 2/3-phosphoglycerate (2/3-PG), lactate, and pyruvate (*Figure 2D*, Supplementary material online, *Figure S3C*). Moreover, we found a significant increase in ^13^C enrichment of PPP intermediates such as ribose-5-phosphate (R5P), sedoheptulose-7-phosphate (S7P), and 6-phosphogluconate (6-PG) (*Figure 2C*, Supplementary material online, *Figure S3D*). Importantly, no differences were detected in metabolites belonging to other glycolytic branch pathways, such as the hexosamine biosynthesis pathway (HBP) and the serine biosynthesis pathway in csKeap1KO compared with WT hearts (see Supplementary material online, *Figure S3E* and *F*), suggesting that activation of NRF2 drives an increase flux into glycolysis and specifically into the PPP. No changes in ^13^C enrichment were detected in metabolites of the TCA cycle (*Figure 2F*). Consistent with these results, we found a significant increase in gene expression of the glucose transporter *Glut1*, but not *Glut4* (see Supplementary material online, *Figure S4A* and *B*) and of key PPP enzymes, such as *G6pd*, *Pgd*, *Taldo1*, and *Tkt* (see Supplementary material online, *Figure S4C–F*). Taken together, these results show that NRF2 activation in cardiomyocytes drives changes in cardiac glucose metabolism and increases glucose flux into the PPP.

### 3.3 Activation of NRF2 in cardiac-specific Keap1 knockout mice protects the heart from pressure overload-induced cardiac dysfunction

**Figure 3:**
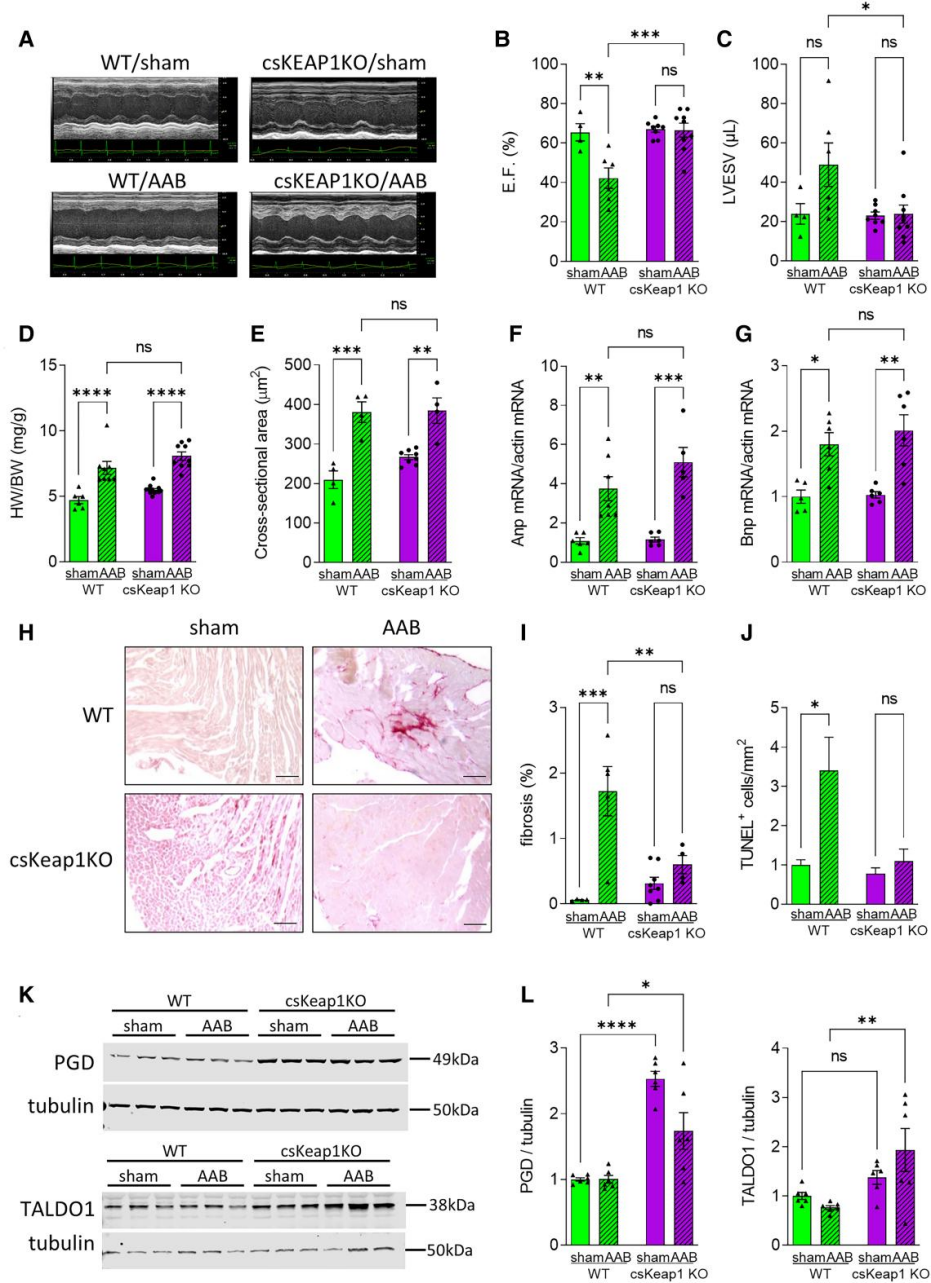
Effects of chronic pressure overload on csKeap1KO hearts. *A*) Representative M-mode echocardiography images of WT and csKeap1KO mice 6 weeks after AAB or sham surgery. *B* and *C*) Ejection fraction (EF) and left ventricular (LV) end-systolic volume (LVESV) in WT and csKeap1KO mice subjected to AAB or sham surgery, *n* ≥ 4/group. *D*) HW/BW, *n* ≥ 6/group. *E*) Cardiomyocyte cross-sectional area measured in heart sections of sham or AAB-operated WT and csKeap1KO hearts, *n* ≥ 4 hearts/group. *F* and *G*) mRNA levels of hypertrophic makers, *Anp* and *Bnp*, *n* ≥ 5/group. *H*) Representative images of heart sections of WT or csKeap1KO mice 6 weeks after sham or AAB surgery, stained with Picrosirius red. Scale bar: 50 µm. *I*) Mean data for fibrosis in heart sections from WT and csKeap1KO mice, *n* ≥ 4 hearts/group. *J*) TUNEL-positive cells per square millimetre of heart tissue in sections of WT and csKeap1KO hearts; *n* ≥ 3 hearts/group. Representative immunoblots (*K*) and quantification data (*L*) for changes in key PPP enzymes 6-phosphogluconate dehydrogenase (Pgd) and Transaldolase1 (Taldo1) in sham or AAB-operated WT and csKeap1KO hearts, *n* = 6. Data are presented as means ± SEM. *P < 0.05, **P < 0.01, ***P < 0.001, ****P < 0.0001 and ns, not significant by two-way ANOVA, followed by Tukey’s multiple comparisons test.

Previous studies have shown that loss of NRF2 exacerbates cardiac dysfunction and promotes HF in settings of stress, such as pressure overload or MI.^8,10,12^ However, whether specific activation of NRF2 in cardiac myocytes (here via Keap1KO) can protect the heart against cardiac dysfunction and failure is unclear. We therefore investigated the role of NRF2 activation under pathological conditions by subjecting csKeap1KO mice and WT littermates to suprarenal abdominal aortic constriction (AAB) or sham surgery. WT mice developed significant cardiac dysfunction compared with sham-operated mice 6 weeks after AAB surgery, as assessed by echocardiography (*Figure 3A–C*). csKeap1KO mice were protected against cardiac dysfunction as evidenced by a lack of reduction in ejection fraction and fractional shortening in comparison with sham-operated csKeap1KO mice (*Figure 3A–C*, Supplementary material online, *Figure S5G* and *H*). Upon pressure overload, similar levels of cardiac hypertrophy were detected in both WT and csKeap1KO mice as evidenced by a significant increase in both heart and cardiomyocyte size, and increased mRNA expression levels of hypertrophic markers, such as atrial natriuretic factor (ANF) and brain natriuretic peptide (BNP; *Figure 3D–G*). However, WT hearts had higher levels of interstitial fibrosis and cardiomyocyte cell death after pressure overload-induced stress than csKeap1KO hearts, as measured by Picrosirius red and TUNEL staining, respectively (*Figure 3H–J*). Overall, our data showed that cardiomyocyte-specific activation of NRF2 via knockout of Keap1 protects the heart against pressure overload-induced cardiomyocyte cell death, cardiac fibrosis, and contractile dysfunction but without significantly affecting the extent of hypertrophy. In line with constitutive activation of NRF2, we found that the mRNA expression levels of genes regulating antioxidant responses (*Nqo1*, *Gsta2*, *Txnrd1*), glutathione reduction (*Gsr*) and synthesis (*Gclc*, *Gclm*), as well as glutathione levels were increased in csKeap1KO compared with WT hearts both under sham and AAB-operated conditions (see Supplementary material online, *Figure S5A–F*). We also assessed levels of PPP enzymes and found upregulation at protein level of some of the PPP-regulating enzymes, particularly PGD and TALDO1 in csKeap1KO hearts compared with WT littermates upon pressure overload (*Figure 3K* and *L*). Overall, these data indicate that the beneficial effects of NRF2 activation are associated with an upregulation NRF2 targets that notably include PPP enzymes.

### 3.4 NRF2 promotes cardiomyocyte survival via activation of the PPP

**Figure 4:**
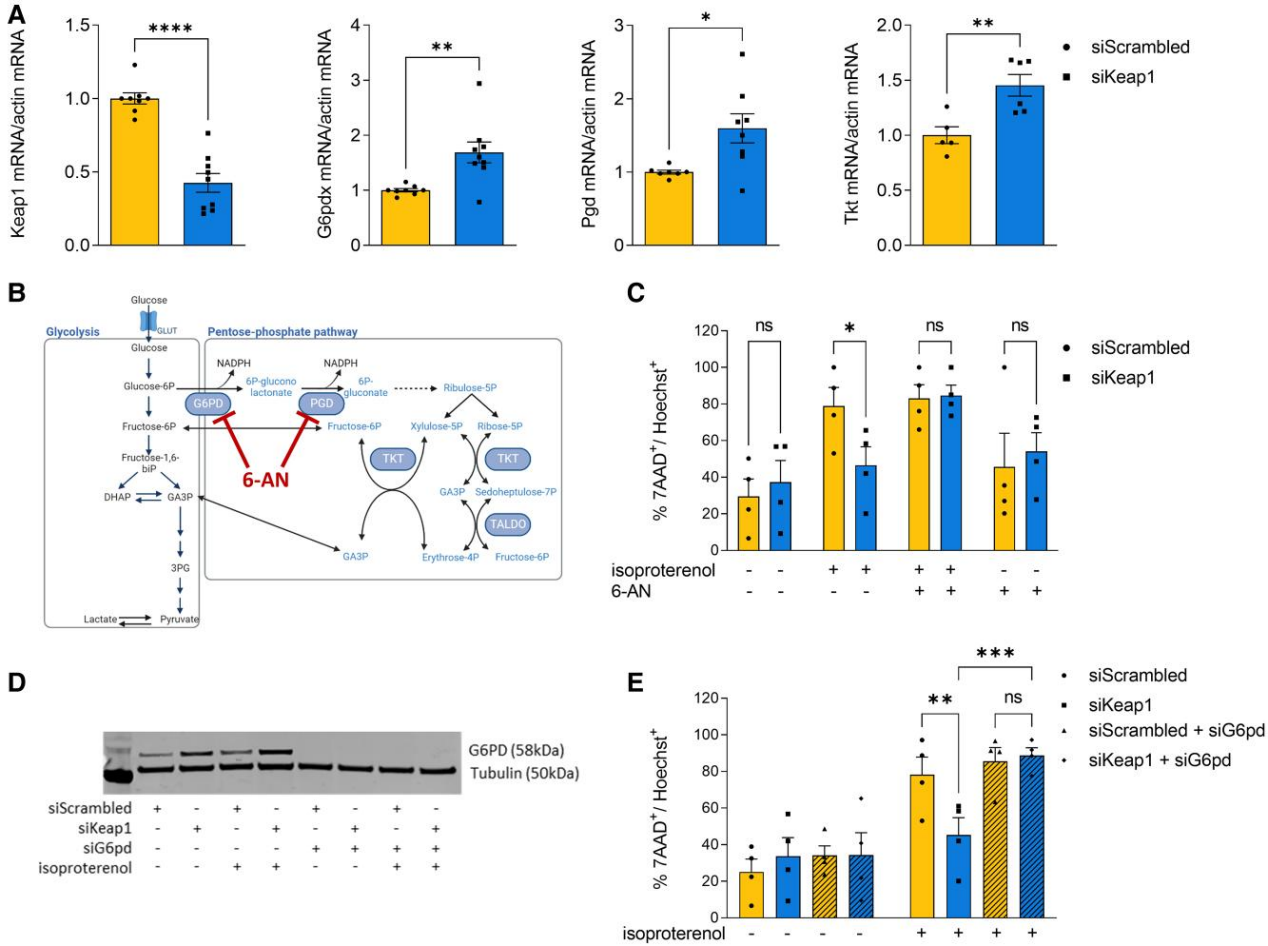
NRF2-mediated upregulation of the PPP prevents stress-induced cardiomyocyte death. *A*) mRNA levels of *Keap1*, *G6pd*, *Pgd*, and *Tkt* measured in NRVMs transfected with either control scrambled siRNA or siKeap1. *n* ≥ 5/group. *B*) Schematic representation of glycolysis and PPP highlighting the PPP enzymes (G6PD and PGD) inhibited by 6-AN. *C*) Cell viability measured as the percentage of 7AAD-positive over Hoechst-positive cells (7AAD^+^/Hoechst^+^) in NRVM transfected with siScrambled or siKeap1, untreated or treated for 24 h with different combinations of isoproterenol and/or 6-AN. Four independent experiments, *n* ≥ 550 cells/group. *D*) Representative immunoblot showing efficient knockdown of G6PD in NRVM transfected with siG6pd alone or in combination with siKeap1. Tubulin was used as a loading marker. *E*) Percentage of 7AAD^+^/Hoechst^+^ calculated in NRVMs transfected with siScrambled or siKeap1 and with or without siG6pd, and treated as indicated; *n* = 4, ≥600 cells. Data are presented as mean ± SEM. *P < 0.05, **P < 0.01, ***P < 0.001 and ****P < 0.0001 and ns, not significant by unpaired Student’s *t*-test or by two-way ANOVA, followed by Tukey’s multiple comparisons test.

To assess the contribution of NRF2-driven alterations in cardiac glucose intermediary metabolism and activation of the PPP to its protective effects in the heart, we next turned to studies in cultured cardiomyocytes. We used a high dose of isoproterenol (100 µmol/L) to induce robust cell death in cultured NRVM (see Supplementary material online, *Figure S6A*) and to investigate its effects on the viability of NRVM in which the levels of Keap1 were manipulated (see Supplementary material online, *Figure S6D* and *F*). In line with our *in vivo* data, we found that the siRNA-mediated knockdown of Keap1 to activate NRF2 in cardiomyocytes resulted in a significant increase in the expression levels of PPP enzymes, including *G6pd*, *Pgd*, and *Tkt* (*Figure 4A*) as well as increased NRF2 levels and of other well-established NRF2 targets (see Supplementary material online, *Figure S6B* and *C*, *E*–*G*). These effects were abolished when NRF2 was also knocked down (see Supplementary material online, *Figure S7*), confirming that the effects of Keap1 knockdown are mediated by NRF2. Of note, we did not detect effects on other cell processes described to be regulated by Keap1 independent of NRF2^36–38^ (see Supplementary material online, *Figures S6D*, *H* and *S8*). We observed that activation of NRF2 by silencing of Keap1 resulted in a significant reduction in NRVM cell death under isoproterenol stimulation when compared with treatment with a scrambled siRNA control (*Figure 4C*). To test whether this effect was specifically mediated by activation of the PPP, NRVMs were treated with 6-AN (500 µmol/L), a selective inhibitor of G6PD and PGD^39^ (*Figure 4B*). Our results show that the beneficial effect of Keap1 silencing on isoproterenol-induced cell death was abolished in the presence of the PPP inhibitor 6-AN (*Figure 4C*). In keeping with this finding, we also found that genetic inhibition of the PPP via silencing of G6PD abolished the protective effect of NRF2 activation (*Figure 4D* and *E*). Taken together, these results indicate that NRF2-mediated activation of the PPP prevents stress-induced cell death in cardiomyocytes.

### 3.5 Endogenous NRF2 acts to reduce stress-induced cardiomyocyte DNA damage

**Figure 5:**
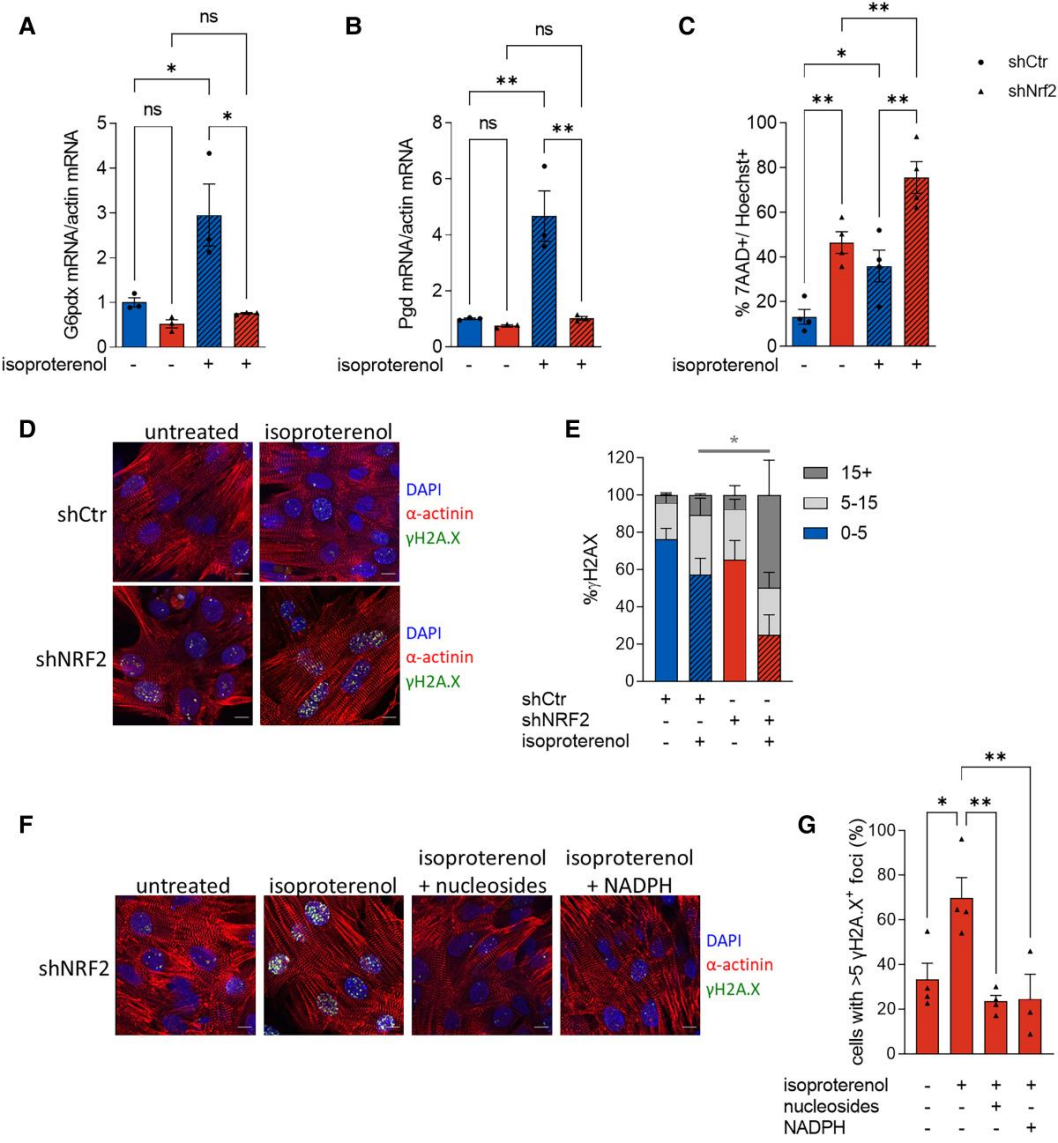
Stress-induced cardiomyocyte DNA damage is limited by NRF2 and the PPP. *A*) mRNA levels of G6pd, and *B*) Pgd in NRVM transduced with adenovirus expressing either control scrambled shRNA or siNrf2 and untreated or treated with isoproterenol. *n* = 5/group. *C*) Quantification of cell viability measured as the percentage of 7AAD^+^/Hoechst^+^ cells. Four independent experiments, *n* ≥ 500 cells/group. *D*) Representative immunofluorescence images of NRVM transduced with adenovirus expressing control shRNA or shNRF2 untreated or treated with 100 µmol/L isoproterenol for 8 h. Cells were stained with antibodies for α-actinin, γ-H2A.X, and DAPI. Scale bar: 10 µm. *E*) Percentage of γ-H2A.X positive cells were divided into three groups depending on the number of γ-H2A.X foci per nucleus: low DNA damage (0–5 foci/nucleus), moderate DNA damage (5–15 foci/nucleus) and high DNA damage (>15 foci/nucleus). NRVM were infected with adenovirus expressing control shRNA or shNRF2, untreated or treated with isoproterenol for 8 h. Four independent biological replicates, *n* ≥ 50 cells per condition. *F*) Representative confocal images of NRVM infected with adenovirus expressing shNRF2 and untreated or treated for 8 h with isoproterenol alone or in combinations with nucleosides or NADPH. Cells were stained with antibodies for α-actinin, γ-H2A.X and DAPI. Scale bar: 10 µm. *G*) Summary and quantification of the percentage of cells with more than five γ-H2A.X positive foci/nucleus. NRVM were infected, treated, and stained as described in (*E*). Three to four biological replicates, *n* ≥ 40 cells/group. Data are presented as mean ± SEM. *P < 0.05, **P < 0.01 and ns, not significant by one-way ANOVA, followed by Tukey’s multiple comparisons test.

We next investigated the mechanism by which the activation of the PPP preserves cell viability in cardiomyocytes upon neurohumoral stress. Several lines of evidence have observed DNA damage associated with stress-induced HF and MI.^40^ An increased level of DNA damage has been shown to exacerbate cardiomyocyte cell death and cardiac dysfunction,^40,41^ and pharmacological or genetic inhibition of DNA damage responses was found to protect the heart against pressure-overload-induced cardiomyocyte hypertrophy.^42^ The PPP regenerates NADPH while converting G6P into R5P, the sugar backbone of nucleotides. Recently, in a cell model of cancer, it was shown that the absence of the tumour suppressor p53 promotes an increase of glucose flux into the PPP which in turn stimulates more efficient DNA damage repair, thus increasing cell survival upon induction of DNA damage with UV irradiation treatment.^43^ In the light of these findings, we assessed whether NRF2-mediated increase in PPP activity promotes cardiomyocyte survival by reducing DNA damage upon stress stimuli. We first examined the changes in PPP enzymes upon isoproterenol treatment in NRVM. Our data showed that isoproterenol treatment induced an increase in the expression level of key PPP enzymes, such as *G6pd* and *Pgd* (*Figure 5A* and *B*), with similar trends also observed for *Taldo1* and *Tkt* (see Supplementary material online, *Figure S9B* and *C*). When NRF2 was silenced (see Supplementary material online, *Figure S9A*), these changes were abolished (*Figure 5A* and *B*, Supplementary material online, *Figure S9B* and *C*), confirming that stress-induced gene expression of the PPP enzymes is regulated by endogenous NRF2 in cardiomyocytes. The treatment of cultured NRVM with isoproterenol induced a significant decrease in cell viability as assessed by the percentage of 7-AAD-positive NRVM. After NRF2 knockdown, isoproterenol treatment resulted in an even higher percentage of 7AAD^+^/Hoechst^+^ NRVM (*Figure 5C*). We then assessed DNA damage levels upon isoproterenol stimulation in NRVM where NRF2 was silenced. Our results showed that isoproterenol treatment induced an increase in the percentage of NRVM nuclei positive for the DNA-damage marker γH2AX. Importantly, we found that this effect was exacerbated in NRVM in which NRF2 was silenced (*Figure 5D* and *E*). Given that the two main products of the PPP are NADPH and the nucleotide precursor R5P, we tested whether exogenous supplementation of either NADPH or nucleosides, as previously described,^43^ was able to rescue the stress-induced DNA damage observed in NRVM-lacking NRF2. Our results showed that supplementation of either NADPH or nucleosides significantly blunted the isoproterenol-induced DNA damage response in NRVM-lacking NRF2 (*Figure 5F* and *G*). In line with these results, we measured U-^13^C-glucose incorporation into nucleotides in NRVM and found a significant decrease in the percentage of ^13^C label enrichment of adenosine diphosphate (ADP), adenosine triphosphate (ATP), uridine diphosphate (UDP), and cytidine triphosphate (CTD) in NRVM-lacking NRF2 when compared with control cells (see Supplementary material online, *Figure S10*). Overall, these data indicate that NRF2-mediated activation of the PPP is required to counteract stress-induced DNA damage responses in cardiomyocytes and that the supplementation of the PPP products, NADPH or nucleotides, reduces the levels of DNA damage.

### 3.6 Activation of NRF2 protects the heart against MI-induced DNA damage and cardiac dysfunction

**Figure 6:**
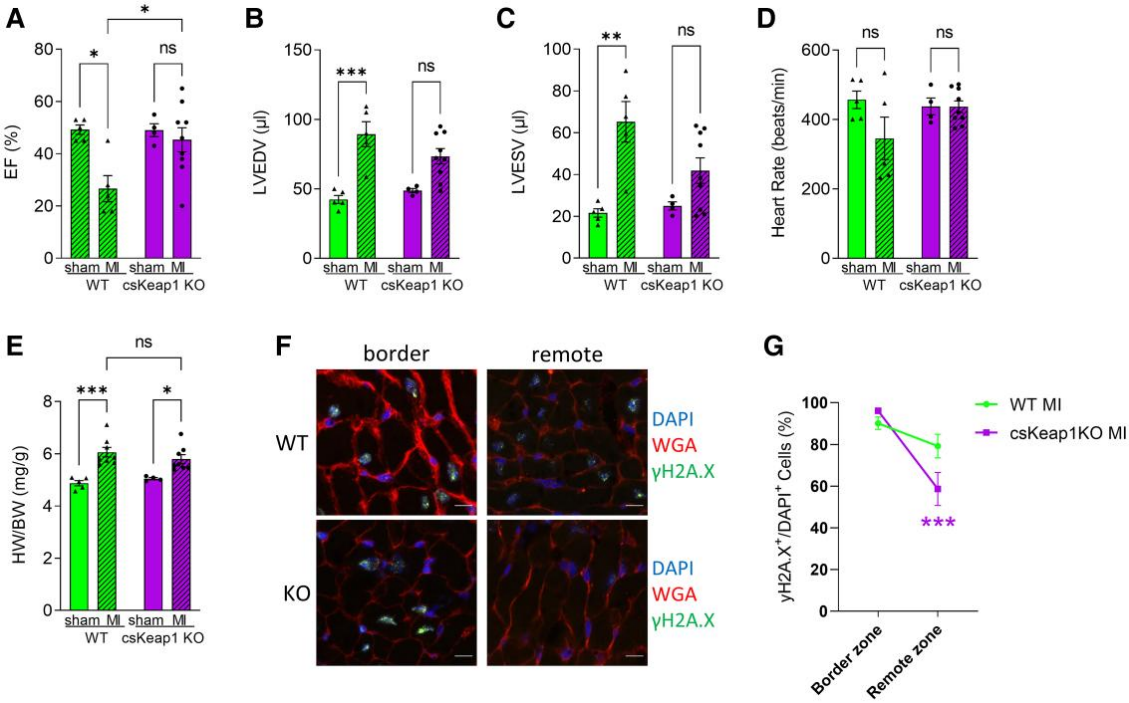
csKeap1KO hearts show better function and less DNA damage after MI. *A–D*) Echocardiography data for EF, LV end-diastolic volume (LVEDV), LV end-systolic volume (LVESV) and heart rate (HR) in WT and csKeap1KO mice subjected to MI or sham surgery; *n* ≥ 5/group]. *E*) HW/BW ratio values for WT and csKeap1KO mice, 4 weeks after MI, *n* = 8/group. *F*) Representative confocal images of cardiac sections from remote or border zone of csKEAP1KO or WT heart after MI and stained with WGA, DAPI, and for γ-H2A.X. *G*) Percentage of γ-H2A.X positive cardiomyocytes in the border zone or remote zone; *n* = 5 hearts/group. Data are presented as mean ± SEM. *P < 0.05, **P < 0.01, ***P < 0.001 and ns, not significant by two-way ANOVA, followed by Tukey’s multiple comparisons test.

To test whether the activation of NRF2 reduces stress-induced cardiomyocyte DNA damage levels *in vivo*, we studied experimental MI which is known to induce significant cardiomyocyte DNA damage both in infarcted and in remote myocardium.^40^ csKeap1KO mice and WT littermates were subjected to permanent LAD coronary artery ligation or sham operation. Four weeks after surgery, echocardiography data showed that csKeap1KO mice had significantly better cardiac function than WT littermates (*Figure 6A–D*, Supplementary material online, *Figure S11A–C*) but a similar increase in heart weight/body weight (HW/BW) ratio (*Figure 6E*). After LAD ligation, csKeap1KO mice and WT littermates showed a similar infarct size and equivalent increases in cardiac fibrosis (see Supplementary material online, *Figure S11D* and *E*). When we looked at the expression levels of PPP enzymes in these experimental conditions, we confirmed an increase in mRNA and protein levels of some of the key PPP enzymes including *G6pd*, *Pdg*, and *Taldo1* in csKeap1KO hearts post-MI when compared with WT littermates (see Supplementary material online, *Figure S12A–C*). We performed immunohistochemistry on tissue sections from the infarcted area (border zone) or the remote zone (at least 2 mm away from the infarct area) of csKeap1KO hearts and WT littermates that underwent LAD permanent ligation, to assess DNA damage. The percentage of γH2AX-positive cardiomyocytes was similarly high in the infarct region of csKeap1KO and WT hearts (*Figure 6F* and *G*). However, we observed a significant reduction of γH2AX-positive cells in the remote zone of csKeap1KO compared with WT hearts 4 weeks after MI (*Figure 6F* and *G*). These data show that activation of NRF2 protects the heart against MI-induced cardiomyocyte DNA damage and dysfunction *in vivo*.

## 4. Discussion

In this study, we identify NRF2 as a key regulator of metabolic adaptation in the heart. Our data reveal for the first time that activation of cardiac NRF2 induces metabolic changes in the heart. We show, using complementary *ex vivo* and *in vitro* approaches, that NRF2 stimulates the diversion of glucose into pathways of intermediary metabolism, in particular, the PPP, which mitigates cardiac dysfunction after stress by generating both NADPH and nucleotides to prevent stress-induced DNA damage and cardiomyocyte death. Using state-of-the-art ^13^C_6_-glucose metabolomics stable-isotope tracing in *ex vivo* experiments, we show that cardiac-specific activation of NRF2 leads to an increased flux of glucose carbon into the PPP but no change in other branch pathways of glycolysis or in glucose carbon flux into the TCA cycle.

NRF2 is a pivotal transcription factor that is known to regulate antioxidant responses and cell homeostasis by controlling the expression of various antioxidant, detoxification, and cytoprotective molecules. Previous studies have shown that a lack of NRF2 exacerbates pathological remodelling and cardiac dysfunction in response to a variety of disease stresses, including pressure overload,^8,10^ MI,^12^ and ischaemia reperfusion.^44^ These cardioprotective effects of NRF2 have been attributed largely to its antioxidant and detoxification effects. Indeed, lack of NRF2 is associated with impaired antioxidant gene expression and increased oxidative stress,^8,10^ a key feature of cardiac pathologies such as HF.^4^ However, in other tissues, NRF2 is described to regulate not only cellular redox homeostasis but also metabolic reprogramming.^14,45–47^ For example, in cancer cells, NRF2 regulates the transcription of key PPP enzymes, including G6PD, PGD, TALDO1, and TKT, together with the glucose transporter GLUT1, other metabolic enzymes, such as malic enzyme 1 (ME1) and isocitrate dehydrogenase 1 (IDH1), and genes regulating *de novo* nucleotide synthesis, such as phosphoribosyl pyrophosphate amidotransferase and methylenetetrahydrofolate dehydrogenase 2.^14,48^ Importantly, the NRF2-mediated expression of PPP enzymes was shown to promote diversion of glucose flux into the PPP, leading to an increase in NADPH regeneration and purine biosynthesis, and ultimately supporting tumour growth.^14^ Indeed, loss-of-function mutations in the *Keap1* gene or gain-of-function mutations in the NRF2 gene have been associated with several types of cancer.^49,50^ Recently, loss of *Keap1* in a model of KRAS-mutant lung adenocarcinoma was shown to activate the PPP and accelerate lung tumorigenesis, while pharmacological inhibition of the PPP with 6-AN reduced tumour growth *in vivo*.^45^ Overall, these recent findings describe an important new role for NRF2 as a key regulator of metabolic reprogramming and intermediary metabolism during stress.^13^ Changes in energy metabolism are a well-recognized feature of pathological cardiac hypertrophy and HF. Healthy adult hearts preferentially utilize fatty acids for energy production; however, in pathological hypertrophy, the heart decreases its capacity for fatty acid oxidation and increases glucose utilization.^19–21^ Less attention, however, has been given to other aspects of metabolic reprogramming in the heart. Intermediary metabolism pathways are essential for the generation of metabolic intermediates required to enhance stress resistance (e.g. NADPH, glutathione), provide cellular building blocks (e.g. amino acids, nucleotides, lipids), and mediate cell signalling [e.g. UDP-*N*-acetylglucosamine (UDP-GlcNAc), ceramides, TCA cycle intermediates]. Recent evidence suggest that glucose intermediary metabolism is altered and may play a role in cardiac pathological settings. For example, increased activity of the HBP, a branch pathway of glycolysis that generates UDP-GlcNAc which is required for the post-translational modification of proteins, has been observed in response to several cardiac stress conditions and linked to both maladaptive and adaptive outcomes.^51^ Controversial results were also obtained from studies aiming at understanding the role of PPP in cardiac injury, with some evidence reporting a beneficial role of PPP activation^52,53^ and others suggesting detrimental effects.^54,55^ It should be noted that most of these studies assessed the role of PPP only by direct modulation of the activity of G6PD. Overall, despite increasing recognition of the role of intermediary metabolism in the heart, little knowledge is available as to how the activation of cardiac glycolytic branch pathways, such as the PPP influences the response to pathological stresses.

To investigate the contribution of NRF2 to regulating cardiac metabolism, we generated csKeap1KO mice, as a novel *in vivo* model of constitutively active NRF2 signalling and uncovered an important functional role of NRF2-mediated regulation of glucose intermediary metabolism, involving the activation of PPP.

First, our *in vivo* data showed that the activation of NRF2 mitigates cardiac dysfunction both after chronic haemodynamic overload and chronic MI. In both settings, NRF2 activation improves cardiac function without altering the extent of hypertrophy *per se*, and upon pressure overload, it reduces cardiomyocyte death and interstitial fibrosis.

Secondly, our data reveal that in addition to the expected increase in the expression of canonical NRF2 targets, increased GSH/GSSG ratio and reduced ROS levels, csKeap1KO hearts display increases in the expression levels of key PPP enzymes at baseline and upon pressure overload or MI. Similarly, in complementary studies in NRVM, the silencing of Keap1 increases the expression of PPP enzymes in a NRF2-dependent manner and reduces isoproterenol-induced cell death.

Thirdly, we find that isoproterenol stress itself increases the expression of PPP enzymes via endogenous NRF2 (*Figure 5*). While this response is insufficient to fully inhibit cell death, the extent of cell death is reduced since the silencing of NRF2 markedly increases isoproterenol-induced cell death.

Fourthly, we show that NRF2-mediated activation of the PPP is necessary to prevent stress-induced cardiomyocyte DNA damage and cell death. As such, pharmacological or genetic inhibition of the PPP abolishes the protective effects of NRF2 in NRVM. Also, the activation of NRF2 and increased expression of PPP enzymes is associated with decreased level of DNA damage in remote myocardium after MI. DNA damage is associated with pathological remodelling and HF;^40–42^ however, a link between activation of the PPP and DNA damage levels has not previously been shown in cardiomyocytes or the heart. Increased PPP activity is a hallmark of many types of cancer, as it plays a crucial role in providing NADPH and nucleotide precursors to sustain cancer cell survival and proliferation.^56^ In a recent cancer study, p53 was shown to mediate glucose carbon diversion into the PPP, resulting in increased *de novo* nucleotide production and increased DNA damage repair and survival.^43^ Similarly, in the present study, we found that the activation of the PPP correlates with decreased levels of DNA damage upon stress stimuli in NRVM and *in vivo*. Interestingly, supplementing NRVM with either NADPH or nucleotides, the two main products of the PPP, can decrease the level of stress-induced DNA damage in cardiomyocytes where NRF2 is absent. Additional studies will be required to establish the signalling mechanisms by which supplementation with NAPDH or nucleotide reduces DNA damage in cardiac cells, and whether the activation of PPP is associated with the activation of DNA damage repair pathways.

Our study has some limitations. We used the silencing of Keap1 as an approach to increase NRF2 activation. Keap1 can have effects that are independent of NRF2 and while we did not find evidence of effects on p62 or cytoskeleton remodelling, we cannot rule out an involvement of other mechanisms. The studies of LAD ligation were performed in female mice since male C57BL6 are highly prone to post-MI rupture,^57^ while pressure overload studies were performed in male mice. It would have been ideal to include both sexes in both experimental models. However, our data show that upregulation of NRF2 protects the heart from cardiac dysfunction without affecting hypertrophic remodelling in both models (male/AAB and female/LAD), suggesting that this protective effect is independent of sex.

Our findings may have clinical implications as selective activation of the PPP may be considered as a novel therapeutic strategy to mitigate cardiac dysfunction after pathological cardiac stress or injury. From this perspective, G6PD may represent an interesting target as recently, AG1, a small molecule that increases the activity of the WT, or mutant variants of G6PD has been discovered.^58^ Further studies will be necessary to test the effects of this small molecule in cardiac pathological stress and HF. NRF2 is described to potentially affect several other metabolic processes, for example, mitochondrial biogenesis^59,60^ and lipid metabolism;^61,62^ hence, future work is also needed to define the relative role of PPP activation vs. other metabolic effects. Additionally, NRF2 may interact with transcription factors, such as ATF4,^63,64^ which may itself exert metabolic effects.^65,66^ The synergistic actions of these (and other) transcription factors in mediating metabolic reprogramming in the heart therefore also merit attention.

**Translational perspective**

Heart failure (HF) continues to be a major cause of morbidity and mortality globally. Pathological cardiac hypertrophy and HF involve metabolic alterations that often precede cardiac dysfunction. While energy metabolism changes are documented, the role of intermediary metabolism is unclear. Currently, there are no strategies specifically aimed at modulating cardiac metabolism. Here, we show that activating nuclear factor erythroid–derived 2-like 2 (NRF2) in the heart increases glycolysis and glucose flux into the pentose phosphate pathway (PPP). This NRF2-mediated PPP activation has cardioprotective effects, preventing stress-induced cardiomyocyte DNA damage and cell death.

## Supplementary Material

cvae250_Supplementary_Data

## Data Availability

The authors declare that the main data supporting the findings of this study are available within the article and in its Supplementary material. The metabolomics data are available upon reasonable request to the corresponding author.
