# Supplementary material for: NRF2 activation in the heart induces glucose metabolic reprogramming and reduces cardiac dysfunction via upregulation of the pentose phosphate pathway
Source: Cardiovasc Res. 2024 Dec 6;121(2):339–52. doi: 10.1093/cvr/cvae250 (PMC12012450; doi:10.1093/cvr/cvae250)

**SUPPLEMENTARY FIGURES**

**
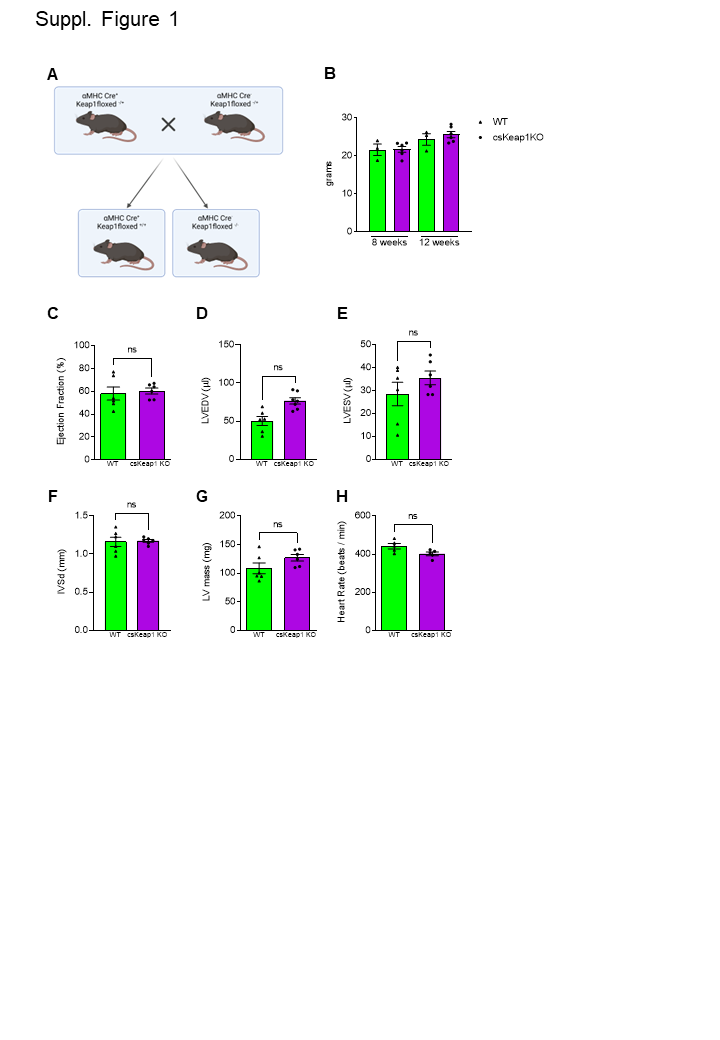
**

**Supplementary Figure 1.**

**(A)** Schematic representation of the breeding strategy. αMHC-Cre positive mice / Keap1 floxed heterozygous mice (αMHC-Cre^+^/Keap1 floxed^+/-^) were crossed with αMHC-Cre negative/Keap1 floxed heterozygous (αMHC-Cre^-^/Keap1 floxed^+/-^). αMHC-Cre^+^/Keap1 floxed^+/+^ (csKeap1KO) were compared to αMHC-Cre^-^/Keap1 floxed^-/-^ mice (WT). **(B)** Weight (grams) of csKeap1KO and WT mice at 8 and 12 weeks of age, n≥3/group, males and females. **(C-H)** Echocardiography parameters and LV mass in csKeap1KO and WT littermates at 12 weeks of age. LV end-diastolic volume, LVEDV; LV end-systolic volume, LVESV; interventricular septal thickness at end diastole, IVSd. n≥6/group. Data are presented as mean ± SEM. *P<0.05, **P < 0.01, ***P < 0.001, and ns, not significant by unpaired Student’s t test.


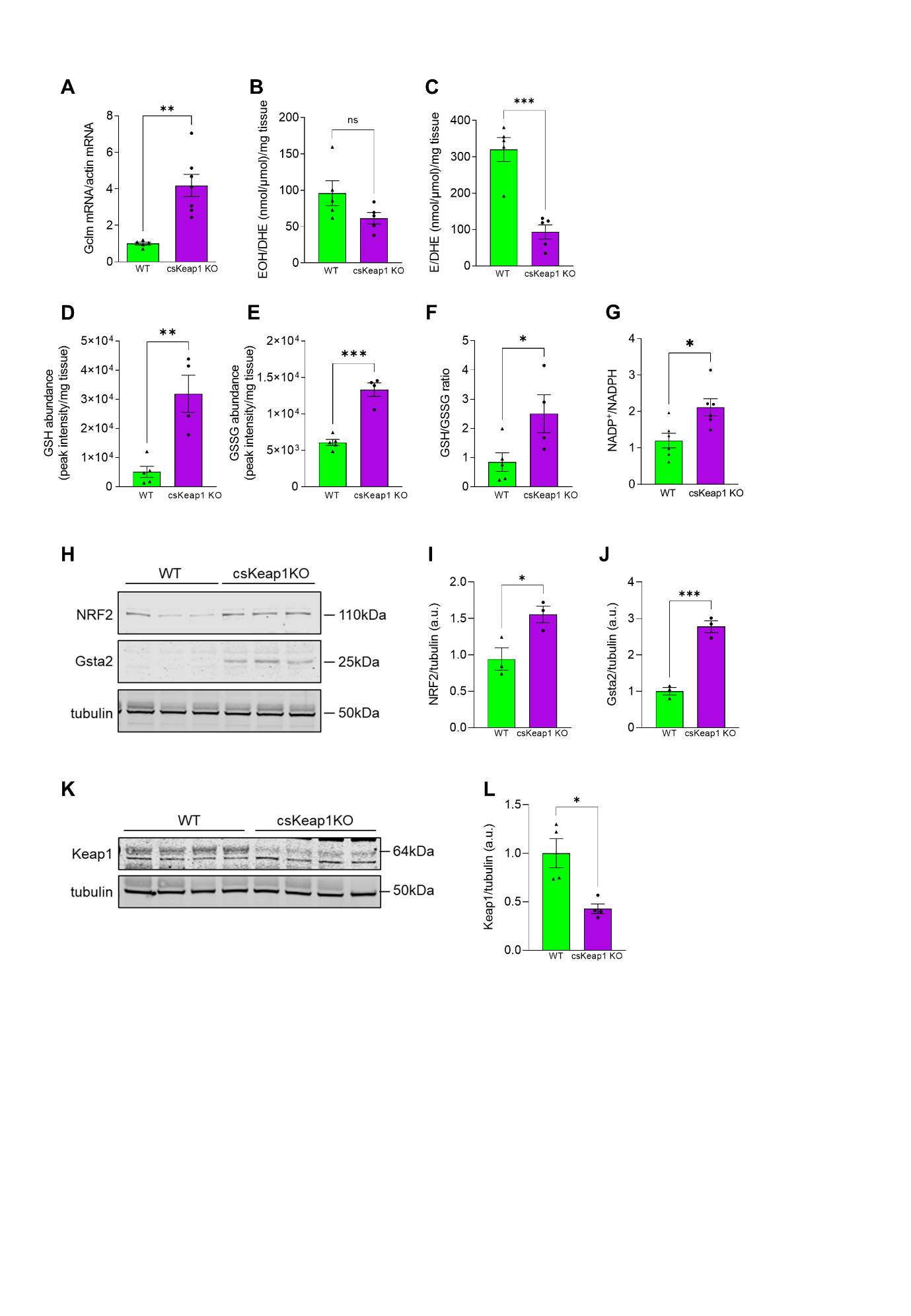


**Supplementary Figure 2.**

**A)** mRNA expression levels of glutamate-cysteine ligase modifier subunit (*Gclm*) in WT vs csKeap1KO hearts, n≥5/group. HPLC detection of Reactive oxygen species-products hydroxyethidium (EOH) **(B)** and ethidium (E) **(C)** per dihydroethidium (DHE) consumed in WT and csKeap1KO hearts (n≥5/group). Levels of reduced (GSH) **(D)**, oxidized (GSSG) **(E)**, and GSH/GSSG ratio **(F)** in WT and csKeap1KO hearts measured by LC-MS, n≥4/group. **G)** NADP+/NADPH ratio measured in freeze-clamped in WT and csKeap1KO hearts, n≥5/group. Representative immunoblots **H, K)** and quantification data **(I-J, L)** for changes in NRF2, Gsta2, and Keap1 in WT and csKeap1KO hearts, N=3-4. Data are presented as mean ± SEM. *P<0.05, **P < 0.01, ***P < 0.001, and ns, not significant by unpaired Student’s t test.

**
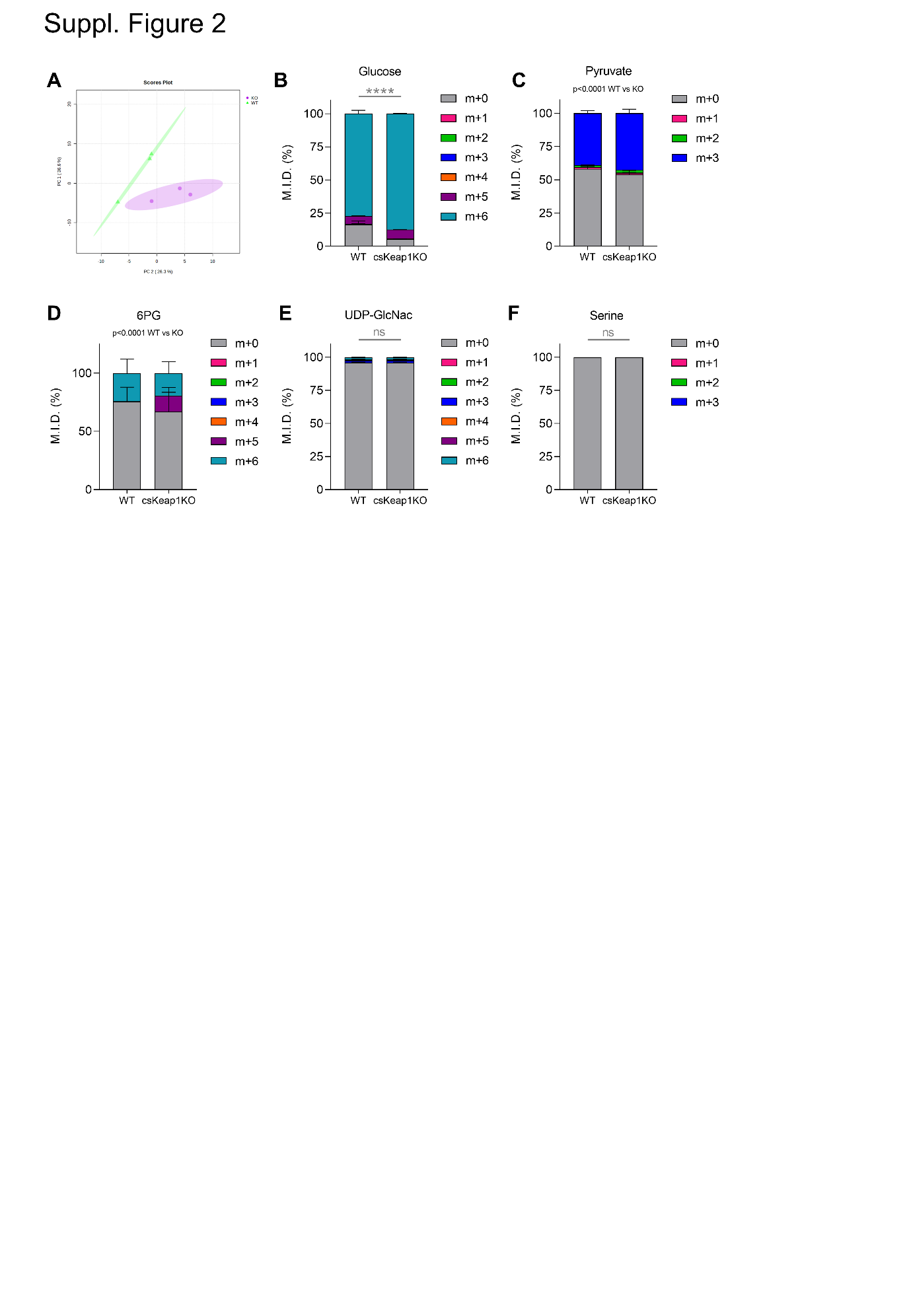
**

**Supplementary Figure 3.**

**A)** Principal component analysis of ^13^C enrichment of metabolites following *ex vivo* Langendorff perfusion of [U-^13^C]glucose in WT and csKeap1KO mouse hearts. N=3/group. ^13^C-glucose incorporation into Glucose **(B)**, Pyruvate **(C)**, 6-phosphogluconolactone (6PG) **(D)**, UDP-GlcNac **(E)**, and Serine **(F)** (Mass Isotopologue Distribution, M.I.D.). Data are presented as mean ± SEM. *P < 0.05, **P < 0.01, ***P < 0.001, ****P<0.0001 and ns, not significant by one-way ANOVA followed by Bonferroni multiple comparison test for mass isotopologue and genotype comparisons.


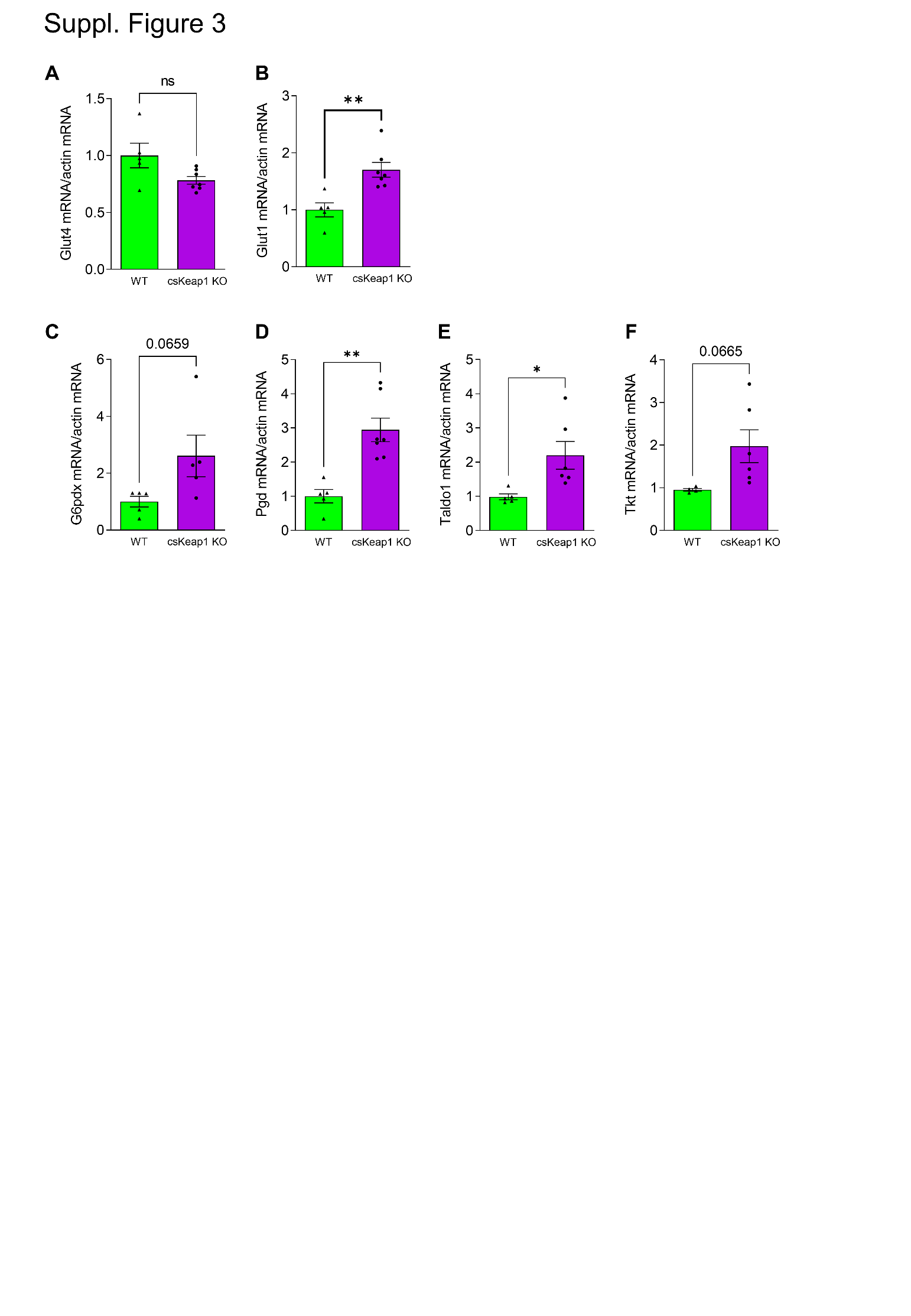


**Supplementary Figure 4.**

**A)** qPCR analysis of Glucose transporter 4 (*Glut4*), **B)** Glucose transporter 1 (*Glut1*), **C)** Glucose-6-phosphate dehydrogenase (*G6pd*), **D)** 6-Phosphogluconate dehydrogenase (*Pgd*), **E)** Transaldolase 1 (*Taldo1*) and **F)** Transketolase (*Tkt*) transcripts; n≥5/group. Data are presented as mean ± SEM. *P < 0.05, **P < 0.01, and ns, not significant by unpaired Student’s t test.

**
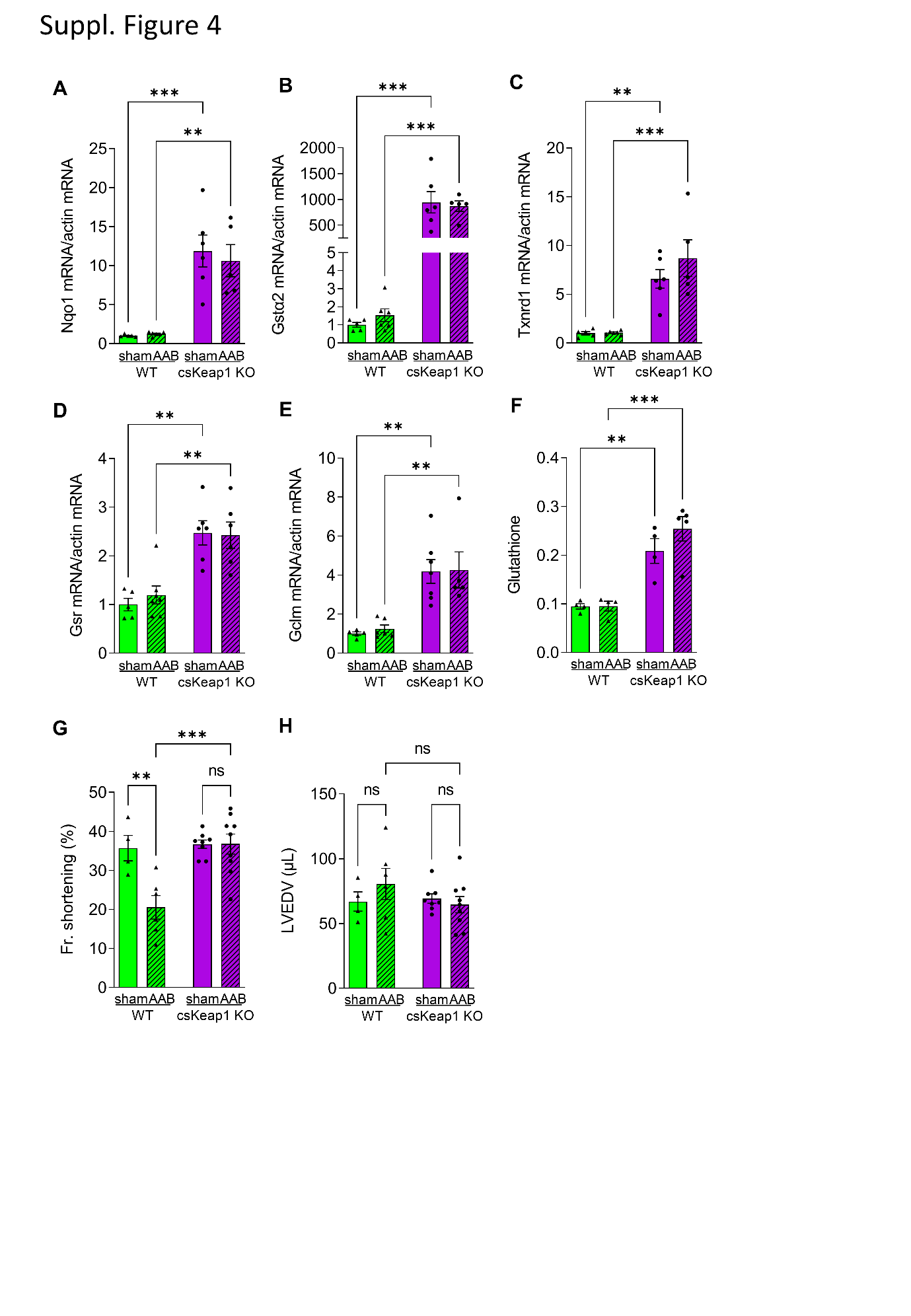
**

**Supplementary Figure 5.**

mRNA levels of NRF2 targets *Nqo1* **(A)**, Gstα2 **(B)**, *Txnrd1* **(C)**, *Gsr* **(D)**, *Gclm* **(E)**, in csKeap1KO vs WT after sham or AAB surgery; n≥4/group. **(F)** Glutathione levels measured by NMR in csKeap1KO vs WT after sham or AAB surgery; n≥4/group. **(G-H)** Fractional (Fr.) shortening and LVEDV in csKeap1KO vs WT after sham or AAB surgery; n≥5/group. Data are presented as mean ± SEM. *P < 0.05, **P < 0.01, ***P < 0.001 and ns, not significant by two-way ANOVA, followed by Tukey’s multiple comparisons test.


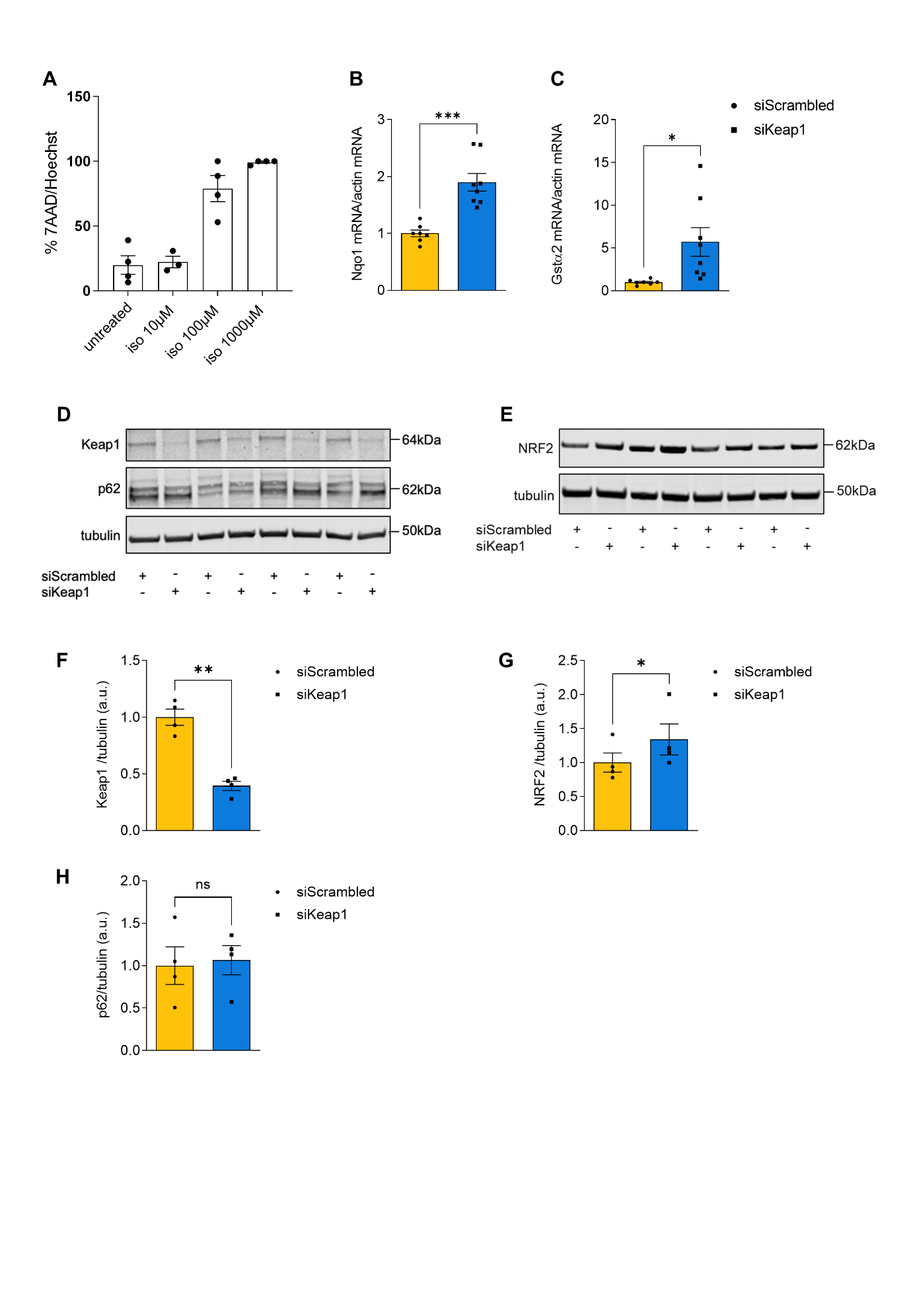


**Supplementary Figure 6.**

**A)** Quantification of cell viability measured as percentage of 7AAD+/Hoechst+ cells in NRVM untreated or treated with increasing concentration of isoproterenol. 4 independent experiments, n≥500 cells/group. qPCR data for *Nqo1* **(B)** and *Gstα2* **(C)** measured in NRVM transfected with either siScrambled control or siKeap. n≥7/group. (**D-E)** Immunoblots and and quantification data **(F-H)** for Keap1, p62, and NRF2 protein levels in NRVM transfected with siScrambled or siKeap1 as indicated, N=4. Data are presented as mean ± SEM. *P <0.05, **P < 0.01, ***P < 0.001 and ns, not significant by Student’s t test.
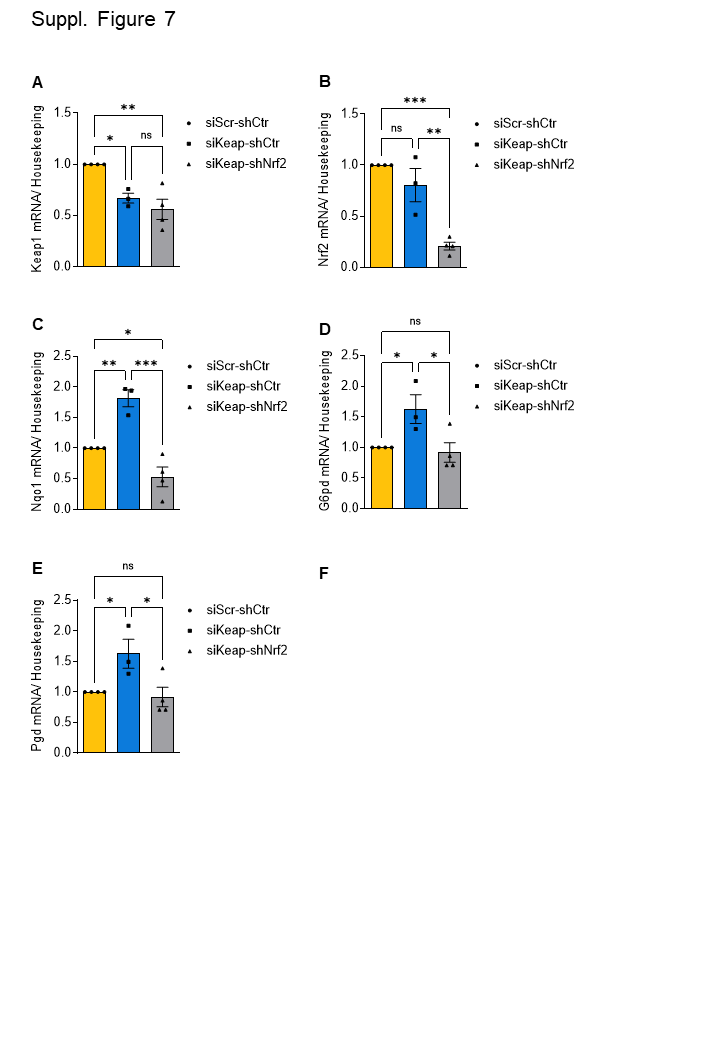


**Supplementary Figure 7.**

qPCR data for *Keap1* **(A)**, *Nrf2* **(B),** *Nqo1* **(C),** *G6pd* **(D),** *Pgd* **(E)** measured in NRVM transfected with either siScrambled control or siKeap, in combination with adenoviral transduction of control shRNA or shNRF2. n≥3/group. Data are presented as mean ± SEM. *P < 0.05, **P<0.01, ***P < 0.001 and ns, not significant by one-way ANOVA followed by Tukey’s multiple comparisons test.


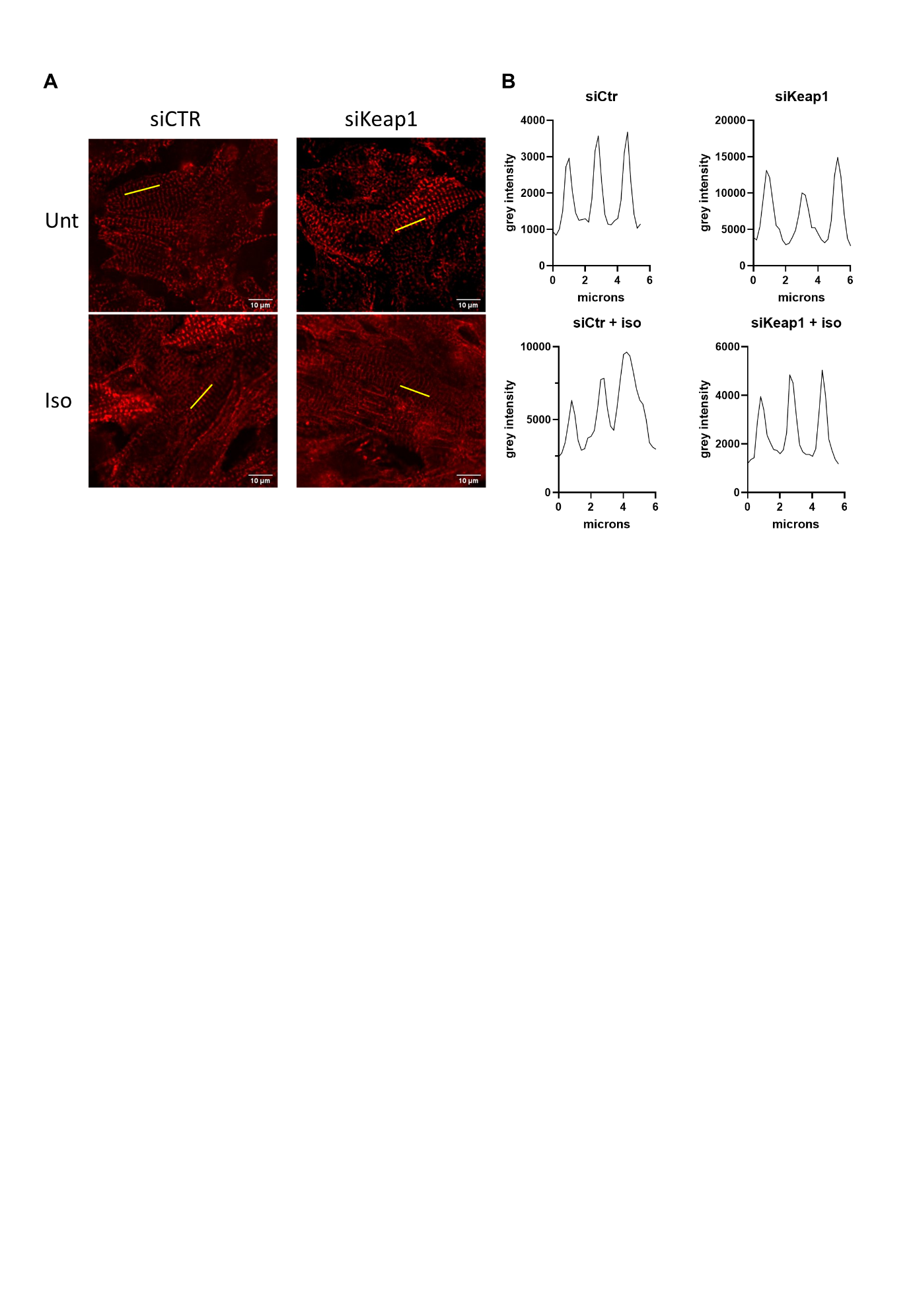


**Supplementary Figure 8.**

**(A)** Representative immunofluorescence images of NRVM transfected with siScrambled or siKeap1, untreated or treated with 100µmol/L isoproterenol for 24h. Cells were stained with antibodies for α-actinin. Scale bar: 10µm. **(B)** Localisation pattern of α-actinin immunofluorescence showing similar pattern in the four different conditions.


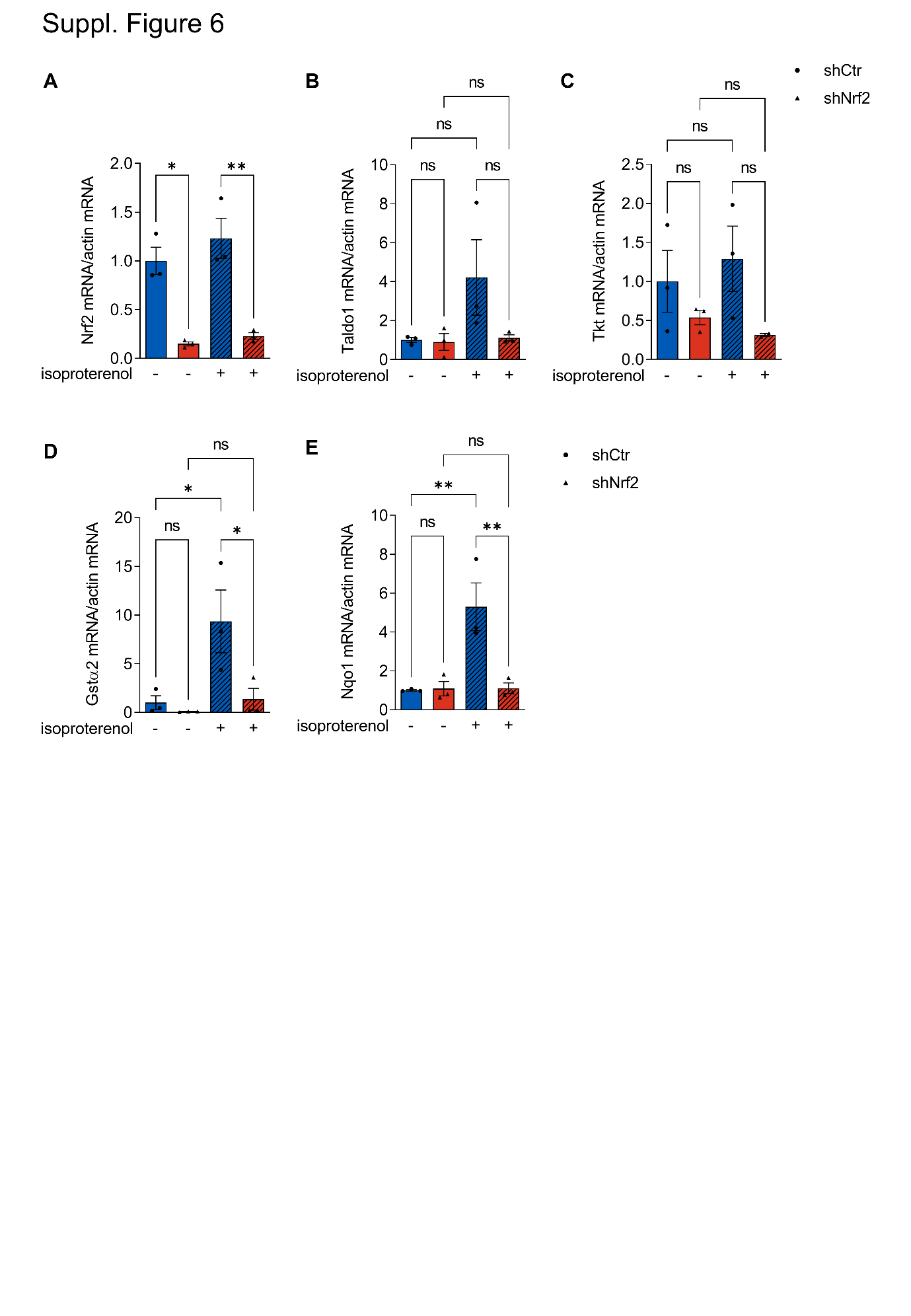


**Supplementary Figure 9.**

mRNA levels of *Nrf2* (**A)**, Transaldolase 1 (Taldo1) **(B)**, Transketolase (Tkt) **(C),** in NRVM infected with Ad.shCtr or Ad.shNrf2 untreated or treated with 100µmol/L isoproterenol for 24h. N=3 biological replicates. Data are presented as mean ± SEM. *P < 0.05, **P < 0.01, and ns, not significant by two-way ANOVA, followed by Tukey’s multiple comparisons test.

**
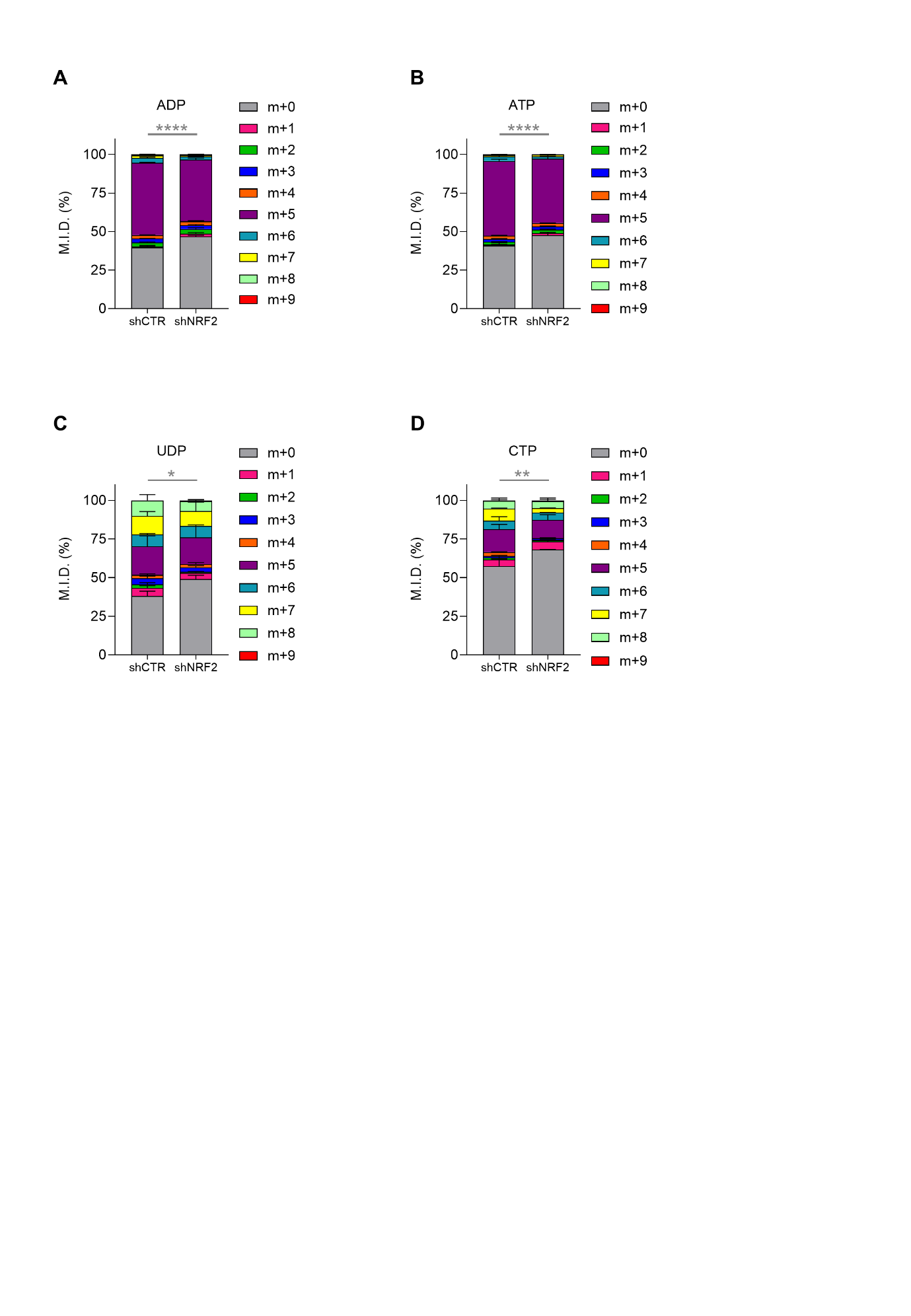
**

**Supplementary Figure 10.**

^13^C-glucose tracing into ADP **(A)**, ATP **(B)**, UDP **(C)**, CTP **(D)** in NRVM transduced with adenovirus expressing control shRNA or shNRF2 and incubated for 18hrs with [U-13C]glucose. N=3 biological replicates. (Mass Isotopologue Distribution, M.I.D.). Data are presented as mean ± SEM. *P < 0.05, **P < 0.01, ****P<0.000 by one-way ANOVA followed by Bonferroni multiple comparison test for mass isotopologue.

**
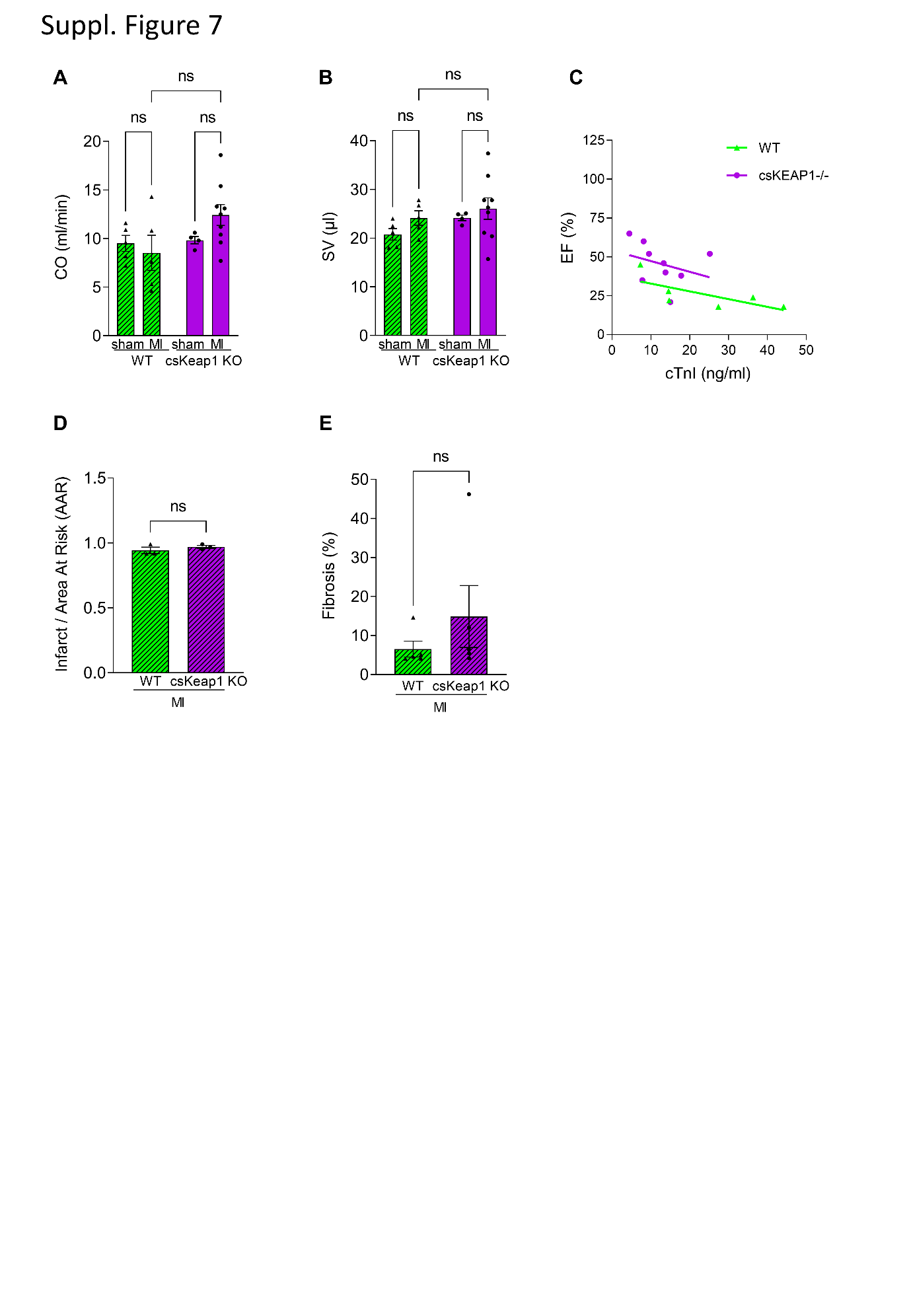
**

**Supplementary Figure 11.**

Echocardiographic parameters in csKeap1KO and WT assessed 4 weeks after sham or LAD ligation surgery; n≥7/group: **A)** Cardiac output, CO; **B)** stroke volume (SV). **(C)** EF related to infarct size as assessed by the plasma cTnI level 2 days after MI. n=6-8/group. For any given infarct size, EF is higher in csKeap1KO mice. **(D)** Infarct size over area at risk assessed by Evans blue staining in WT and csKeap1KO hearts. n=3/group. **(E)** % fibrosis assessed by Picrosirius red staining of heart sections 4 weeks after sham or LAD ligation surgery. n=5/group. Data are presented as mean ± SEM. *P < 0.05, **P < 0.01, ***P < 0.001 and ns, not significant by two-way ANOVA, followed by Tukey’s multiple comparisons test or by unpaired Student’s t test.


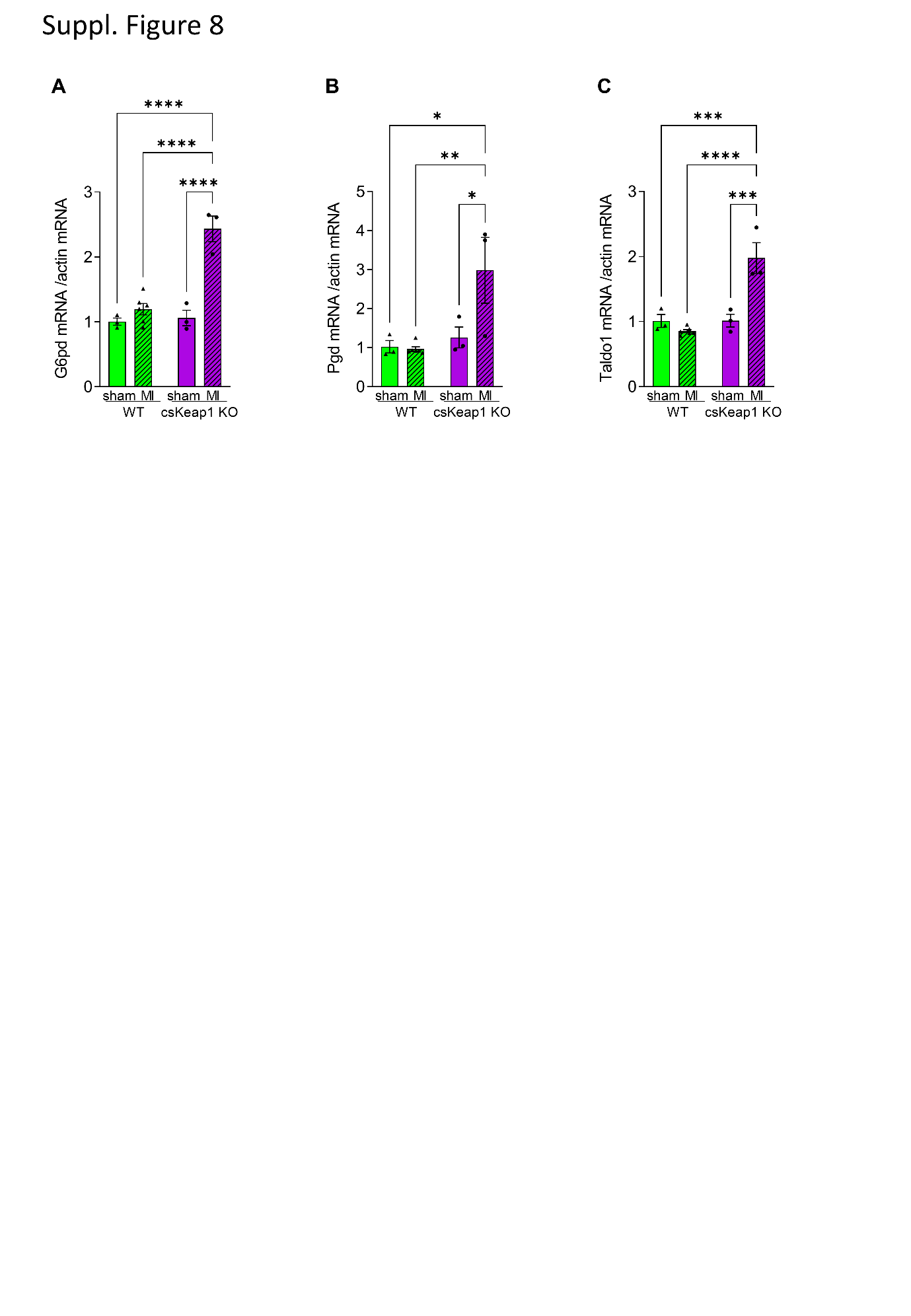


**Supplementary Figure 12.**

mRNA levels of G6pd **(A)**, Pgd **(B)** and Taldo1 **(C)** in WT or csKeap1KO hearts 4 weeks after sham or LAD ligation surgery. n≥3/group. *P < 0.05, **P < 0.01, ***P < 0.001, ****P<0.0001 and ns, not significant by two-way ANOVA, followed by Tukey’s multiple comparisons test.

**SUPPLEMENTARY TABLES:**

| **Name** | **Formula** | **Mass** | **Retention Time** | **HMDB** | **PubChem** | **ChEBI** | **KEGG** | **METLIN** |
| --- | --- | --- | --- | --- | --- | --- | --- | --- |
| **1-Methyl-L-histidine** | C7H11N3O2 | 169.0851 | 6.7 | HMDB0000001 | 92105 | 50599 | C01152 | 3741 |
| **2-Deoxy-D-Glucose** | C6H12O5 | 164.0685 | 2.19 | HMDB0062477 | 108223 | 15866 | NA | NA |
| **2-Hydroxyhexanoic acid** | C6H12O3 | 132.0786 | 1.47 | HMDB0001624 | 99824 | 86542 | NA | 6332 |
| **3-Hydroxy 3-Methyl Glutarate** | C6H10O5 | 162.0528 | 7.55 | HMDB0000355 | 1662 | 16831 | C03761 | 5344 |
| **3-Hydroxy Butyrate** | C4H8O3 | 104.0473 | 3.04 | HMDB0000011 | 92135 | 17066 | C01089 | NA |
| **3-Hydroxy Glutarate** | C5H8O5 | 148.0372 | 8.07 | HMDB0000428 | 181976 | NA | NA | 5417 |
| **3-Hydroxymethylglutarate** | C6H10O5 | 162.0528 | 5.06 | HMDB0000355 | 1662 | 16831 | C03761 | 5344 |
| **3-Methyl-L-histidine** | C7H11N3O2 | 169.0851 | 8.3 | HMDB0000479 | 64969 | 27596 | C01152 | 5466 |
| **3-Phospho-D-glycerol phosphate (1,3 BPG)** | C3H8O10P2 | 265.9593 | 11.26 | HMDB0001270 | 439191 | 16001 | C00236 | NA |
| **6-Phospho D-glucono-1,5-lactone** | C6H11O9P | 258.0141 | 6.03 | HMDB0001127 | 439452 | 16938 | C01236 | 6022 |
| **6-Phosphogluconic acid** | C6H13O10P | 276.0246 | 9.75 | HMDB0001316 | 91493 | 48928 | C00345 | 367 |
| **Acetoacetate** | C4H6O3 | 102.0317 | 2.41 | HMDB0000060 | 96 | 15344 | C00164 | 276 |
| **Acetyl-CoA** | C23H38N7O17P3S | 809.1258 | 8.2 | HMDB0001206 | 444493 | 15351 | C00024 | 6082 |
| **Adenosine** | C10H13N5O4 | 267.0968 | 1.6 | HMDB0000050 | 60961 | 16335 | C00212 | 86 |
| **Adenosine 3-5-cyclic monophosphate (cAMP)** | C10H12N5O6P | 329.0525 | 3.13 | HMDB0000058 | 6076 | 17489 | C00575 | 5120 |
| **Adenosine 5'-diphosphate (ADP)** | C10H15N5O10P2 | 427.0294 | 8.94 | HMDB0001341 | 6022 | 16761 | C00008 | 6175 |
| **Adenosine 5'-monophosphate (AMP)** | C10H14N5O7P | 347.0631 | 8.46 | HMDB0000045 | 6083 | 16027 | C00020 | 5111 |
| **Adenosine 5'-triphosphate (ATP)** | C10H16N5O13P3 | 506.9957 | 9.27 | HMDB0000538 | 5957 | 15422 | C00002 | 5523 |
| **AICAR** | C9H14N4O5 | 258.0964 | 1.97 | HMDB0001517 | 65110 | 18406 | C04677 | 6294 |
| **Cis-Aconitate** | C6H6O6 | 174.0164 | 7.98 | HMDB0000072 | 643757 | 32805 | C00417 | 5130 |
| **Citrate** | C6H8O7 | 192.027 | 9.5 | HMDB0000094 | 311 | 30769 | C00158 | 124 |
| **Citryl-CoA** | C27H42N7O22P3S | 941.1316 | 5.23 | NA | 11966144 | 15459 | NA | NA |
| **Coenzyme A (CoA)** | C21H36N7O16P3S | 767.1152 | 8.4 | HMDB0001423 | 87642 | 15346 | C00010 | NA |
| **Creatine** | C4H9N3O2 | 131.0695 | 4.94 | HMDB0000064 | 586 | 16919 | C00300 | 7 |
| **Creatinine** | C4H7N3O | 113.0589 | 1.98 | HMDB0000562 | 588 | 16737 | C00791 | 8 |
| **Cyclic guanosine monophosphate (cGMP)** | C10H12N5O7P | 345.0474 | 4.65 | HMDB0001314 | 24316 | 24316 | C00942 | 6152 |
| **Cytidine 5'-diphosphate (CDP)** | C9H15N3O11P2 | 403.0182 | 9.63 | HMDB0001546 | 6132 | 17239 | C00112 | 6313 |
| **Cytidine 5'-triphosphate (CTP)** | C9H16N3O14P3 | 482.9845 | 9.83 | HMDB0000082 | 6176 | 17677 | C00063 | 5136 |
| **Cytidine-5-monophosphate (CMP)** | C9H14N3O8P | 323.0519 | 9.36 | HMDB0000095 | 6131 | 17361 | C00055 | 5143 |
| **D-2-Hydroxyglutarate** | C5H8O5 | 148.0372 | 7.6 | HMDB0000606 | 439391 | 32796 | C01087 | 5581 |
| **D-Erythrose** | C4H8O4 | 120.0423 | 2.85 | HMDB0002649 | 5460672 | 23956 | C01796 | 289 |
| **D-Erythrose 4-phosphate** | C4H9O7P | 200.0086 | 8.85 | HMDB0001321 | 122357 | 48153 | C00279 | 6158 |
| **D-Fructose** | C6H12O6 | 180.0634 | 2.91 | HMDB0000660 | 439709 | 28645 | C02336 | 135 |
| **D-Fructose 1,6-bisphosphate (FBP)** | C6H14O12P2 | 339.996 | 10.9 | HMDB0001058 | 10267 | 28013 | C05378 | 147 |
| **D-Fructose 6-phosphate** | C6H13O9P | 260.0297 | 8.9 | HMDB0000124 | 69507 | 15946 | C00085 | 5159 |
| **D-Galactonate** | C6H12O7 | 196.0583 | 5.48 | HMDB0000565 | 128869 | 16534 | C00880 | 3336 |
| **D-Gluconate** | C6H12O7 | 196.0583 | 6.03 | HMDB0000625 | 10690 | 33198 | C00257 | 345 |
| **D-Glucosamine** | C6H13NO5 | 179.0794 | 5.93 | HMDB0001514 | 439213 | 47977 | C00329 | 266 |
| **D-Glucosamine 6-phosphate** | C6H14NO8P | 259.0457 | 10.09 | HMDB0001254 | 439217 | 15873 | C00352 | 6111 |
| **D-Glucose** | C6H12O6 | 180.0634 | 3.7 | HMDB0000122 | 5793 | 4167 | C00221 | 133 |
| **D-Glucose 1-phosphate** | C6H13O9P | 260.0297 | 8.97 | HMDB0001586 | 3403 | 29042 | C00103 | NA |
| **D-Glucose 6-phosphate** | C6H13O9P | 260.0297 | 9.53 | HMDB0001401 | 5958 | 4170 | C00092 | 145 |
| **D-Glycerate 2-phosphate (2-PG)** | C3H7O7P | 185.9929 | 9.51 | HMDB0003391 | 439278 | 17835 | C00631 | 6915 |
| **D-Glycerate 3-phosphate (3-PG)** | C3H7O7P | 185.9929 | 9.51 | HMDB0000807 | 724 | 17050 | C00597 | 150 |
| **Dihydroxyacetone phosphate (DHAP)** | C3H7O6P | 169.998 | 8.8 | HMDB0001473 | 668 | 16108 | C00111 | 6262 |
| **Dihydroxyphenylalanine (DOPA)** | C9H11NO4 | 197.0688 | 9.4 | HMDB0000609 | 836 | 49168 | C00355 | NA |
| **Dimethyl-glycine** | C4H9NO2 | 103.0633 | 4.07 | HMDB0000092 | 673 | 17724 | C01026 | 277 |
| **D-isocitrate** | C6H8O7 | 192.027 | 7.89 | HMDB0000193 | 1198 | 30887 | C00311 | 3328 |
| **D-Ribose** | C5H10O5 | 150.0528 | 1.97 | HMDB0000283 | 5779 | 47013 | C00121 | 313 |
| **D-Ribose 5-phosphate** | C5H11O8P | 230.0192 | 8.84 | HMDB0001548 | 440101 | 52742 | C00117 | 6315 |
| **D-Ribulose 5-phosphate** | C5H11O8P | 230.0192 | 8.5 | HMDB0000618 | 439184 | 17363 | C00199 | 5591 |
| **D-Sedoheptulose-7-phosphate** | C7H15O10P | 290.0403 | 8.88 | HMDB0001068 | 92042786 | 133983 | C05382 | 5980 |
| **D-Xylulose 5-phosphate** | C5H11O8P | 230.0192 | 8.93 | HMDB0000868 | 439190 | 16332 | C00231 | 5829 |
| **FAD** | C27H33N9O15P2 | 785.1571 | 6.4 | HMDB0001248 | 643975 | 16238 | C00016 | 6106 |
| **Folate** | C19H19N7O6 | 441.1397 | 8.74 | HMDB0000121 | 6037 | 27470 | C00504 | 246 |
| **Fumarate** | C4H4O4 | 116.011 | 8.05 | HMDB0000134 | 444972 | 18012 | C00122 | 3242 |
| **GDP-glucose** | C16H25N5O16P2 | 605.0772 | 8.56 | HMDB0003351 | 644106 | 62254 | C00394 | 6900 |
| **Gluconolactone** | C6H10O6 | 178.0477 | 2.2 | HMDB0000150 | 7027 | 16217 | C00198 | 353 |
| **Glutathione reduced - GSH** | C10H17N3O6S | 307.0838 | 7.63 | HMDB0000125 | 124886 | 16856 | C00051 | 44 |
| **Glutathione oxidized - GSSG** | C20H32N6O12S2 | 612.152 | 10.51 | HMDB0003337 | 65359 | 17858 | C00127 | NA |
| **Glyceraldehyde 3-phosphate (GAP/G3P)** | C3H7O6P | 169.998 | 9.65 | HMDB0001112 | 729 | 17138 | C00118 | 3294 |
| **Glycerate** | C3H6O4 | 106.0266 | 3.95 | HMDB0000139 | 439194 | 32398 | C00258 | 280 |
| **Glycerol** | C3H8O3 | 92.0473 | 2.99 | HMDB0000131 | 753 | 17754 | C00116 | 105 |
| **Glycerophosphocholine** | C8H21NO6P | 258.1106 | 6.88 | HMDB0000086 | 71920 | 16870 | C00670 | 370 |
| **Glycine** | C2H5NO2 | 75.032 | 5.55 | HMDB0000123 | 750 | 15428 | C00037 | 20 |
| **Glyoxylate** | C2H2O3 | 74.0004 | 6.97 | HMDB0000119 | 760 | 16891 | C00048 | 3213 |
| **Guanosine 5'-diphosphate (GDP)** | C10H15N5O11P2 | 443.0243 | 10.01 | HMDB0001201 | 8977 | 17552 | C00035 | 6077 |
| **Guanosine 5'-triphosphate (GTP)** | C10H16N5O14P3 | 522.9907 | 9.85 | HMDB0001273 | 6830 | 15996 | C00044 | 6128 |
| **Guanosine monophosphate (GMP)** | C10H14N5O8P | 363.058 | 9.79 | HMDB0001397 | 6804 | 17345 | C00144 | 6216 |
| **3-Hydroxy-3-methylglutaryl-CoA (HMG-CoA)** | C27H44N7O20P3S | 911.1575 | 9.78 | HMDB0001375 | 439218 | 15467 | C00356 | 6201 |
| **Hydroxy-L-proline** | C5H9NO3 | 131.0582 | 5.51 | HMDB0000725 | 5810 | 18095 | C01157 | 257 |
| **Hydroxypyruvate** | C3H4O4 | 104.011 | 2.6 | HMDB0001352 | 964 | 30841 | C00168 | 482 |
| **Inosine 5'-monophosphate (IMP)** | C10H13N4O8P | 348.0471 | 8.93 | HMDB0000175 | 8582 | 17202 | C00130 | 5196 |
| **Itaconate** | C5H6O4 | 130.0266 | 6.8 | HMDB0002092 | 811 | 30838 | C00490 | 6483 |
| **L-Acetylcarnitine** | C9H18NO4 | 204.1236 | 3.51 | HMDB0000201 | 7045767 | NA | C02571 | 5213 |
| **Lactate** | C3H6O3 | 90.0317 | 2.94 | HMDB0000190 | 107689 | 422 | C00186 | 5205 |
| **L-Alanine** | C3H7NO2 | 89.0477 | 5.13 | HMDB0000161 | 5950 | 16977 | C00041 | NA |
| **L-Anserine** | C10H16N4O3 | 240.1222 | 8.44 | HMDB0000194 | 112072 | 18323 | C01262 | 5209 |
| **L-Arginine** | C6H14N4O2 | 174.1117 | 9.44 | HMDB0000517 | 6322 | 16467 | C00062 | 5502 |
| **L-Asparagine** | C4H8N2O3 | 132.0535 | 5.91 | HMDB0000168 | 6267 | 17196 | C00152 | 14 |
| **L-Aspartate** | C4H7NO4 | 133.0375 | 7.65 | HMDB0000191 | 5960 | 17053 | C00049 | 5206 |
| **L-Aspartyl-4-phosphate** | C4H8NO7P | 213.0038 | 1.43 | HMDB0012250 | 152441 | 15836 | C03082 | NA |
| **L-Carnosine** | C9H14N4O3 | 226.1066 | 7.8 | HMDB0000033 | 439224 | 15727 | C00386 | 38 |
| **L-Citrulline** | C6H13N3O3 | 175.0957 | 6.47 | HMDB0000904 | 9750 | 16349 | C00327 | 16 |
| **L-Cystathionine** | C7H14N2O4S | 222.0674 | 7.98 | HMDB0000099 | 439258 | 17482 | C02291 | 39 |
| **L-Cysteine** | C3H7NO2S | 121.0197 | 8.77 | HMDB0000574 | 5862 | 17561 | C00097 | 5556 |
| **L-Cystine** | C6H12N2O4S2 | 240.0238 | 8.33 | HMDB0000192 | 67678 | 16283 | C00491 | 5207 |
| **L-Glutamate** | C5H9NO4 | 147.0532 | 7.77 | HMDB0000148 | 33032 | 16015 | C00025 | 5174 |
| **L-Glutamine** | C5H10N2O3 | 146.0691 | 5.71 | HMDB0000641 | 5961 | 18050 | C00064 | 5614 |
| **L-Histidine** | C6H9N3O2 | 155.0695 | 6.13 | HMDB0000177 | 6274 | 15971 | C00135 | 21 |
| **L-Homocystine** | C8H16N2O4S2 | 268.0551 | 8.43 | HMDB0000676 | 69382 | 89698 | C01817 | NA |
| **L-Homoserine** | C4H9NO3 | 119.0582 | 5.5 | HMDB0000719 | 12647 | 15699 | C00263 | 5687 |
| **L-Homoserine phosphate** | C4H10NO6P | 199.0246 | 9.83 | HMDB0003484 | 151187 | 15961 | C01102 | NA |
| **L-Hydroxylysine** | C6H14N2O3 | 162.1004 | 11.3 | HMDB0000450 | 3032849 | 18040 | C16741 | 5439 |
| **L-Isoleucine** | C6H13NO2 | 131.0946 | 3.1 | HMDB0000172 | 6306 | 17191 | C00407 | 5193 |
| **L-Leucine** | C6H13NO2 | 131.0946 | 2.8 | HMDB0000687 | 6106 | 15603 | C00123 | 24 |
| **L-Lysine** | C6H14N2O2 | 146.1055 | 12.73 | HMDB0000182 | 5962 | 18019 | C00047 | 5200 |
| **L-Methionine** | C5H11NO2S | 149.051 | 3.3 | HMDB0000696 | 6137 | 16643 | C00073 | 5664 |
| **L-Ornithine** | C5H12N2O2 | 132.0899 | 6.41 | HMDB0000214 | 6262 | 15729 | C00077 | 27 |
| **L-Phenylalanine** | C9H11NO2 | 165.079 | 2.42 | HMDB0000159 | 6140 | 17295 | C00079 | 28 |
| **L-Proline** | C5H9NO2 | 115.0633 | 4.5 | HMDB0000162 | 145742 | 17203 | C00148 | 29 |
| **L-Serine** | C3H7NO3 | 105.0426 | 5.9 | HMDB0000187 | 5951 | 17115 | C00065 | 5203 |
| **L-Threonine** | C4H9NO3 | 119.0582 | 5.96 | HMDB0000167 | 6288 | 16857 | C00188 | 32 |
| **L-Tryptophan** | C11H12N2O2 | 204.0899 | 2.71 | HMDB0000929 | 6305 | 16828 | C00078 | 5879 |
| **L-Tyrosine** | C9H11NO3 | 181.0739 | 3.17 | HMDB0000158 | 6057 | 17895 | C00082 | 34 |
| **L-Valine** | C5H11NO2 | 117.079 | 4 | HMDB0000883 | 6287 | 16414 | C00183 | 5842 |
| **Maleate** | C4H4O4 | 116.011 | 2.57 | HMDB0000176 | 444266 | 18300 | C01384 | 4198 |
| **Malate** | C4H6O5 | 134.0215 | 8.04 | HMDB0000744 | 525 | 6650 | C03668 | 118 |
| **Malonyl-CoA** | C24H38N7O19P3S | 853.1156 | 9.85 | HMDB0001175 | 10663 | 15531 | C03188 | 6056 |
| **N-Acetyl-D-glucosamine (GlcNAc)** | C8H15NO6 | 221.0899 | 3.34 | HMDB0000215 | 439174 | 506227 | C00140 | 3356 |
| **N-Acetyl-D-glucosamine 1-phosphate** | C8H16NO9P | 301.0563 | 8.55 | HMDB0001367 | 7115 | 7125 | C04256 | 6194 |
| **N-Acetyl-D-glucosamine 6-phosphate** | C8H16NO9P | 301.0563 | 8.53 | HMDB0001062 | 440996 | 15784 | C00357 | 5975 |
| **N-Acetyl-L-aspartate** | C6H9NO5 | 175.0481 | 7.85 | HMDB0000812 | 65065 | 21547 | C01042 | 5776 |
| **NAD+** | C21H28N7O14P2 | 664.1169 | 8.28 | HMDB0000902 | 5893 | 44215 | C00003 | 5858 |
| **NADH** | C21H29N7O14P2 | 665.1248 | 7.31 | HMDB0000902 | 5893 | 44215 | C00003 | 5858 |
| **NADP+** | C21H29N7O17P3 | 744.0833 | 10.41 | HMDB0000217 | 5886 | 44409 | C00006 | 5227 |
| **NADPH** | C21H30N7O17P3 | 745.0911 | 9.83 | HMDB0000217 | 5886 | 44409 | C00006 | 5227 |
| **N-Carbamoyl-L-aspartate** | C5H8N2O5 | 176.0433 | 9.22 | HMDB0000828 | 93072 | 15859 | C00438 | 5791 |
| **O-Phospho-L-serine** | C3H8NO6P | 185.0089 | 9.7 | HMDB0001721 | 106 | 37712 | C01005 | 6338 |
| **Oroate** | C5H4N2O4 | 156.0171 | 1.93 | HMDB0000226 | 967 | 16742 | C00295 | 318 |
| **Oxaloacetate** | C4H4O5 | 132.0059 | 9.13 | HMDB0000223 | 970 | 30744 | C00036 | 123 |
| **Oxoglutarate (α-KG)** | C5H6O5 | 146.0215 | 7.1 | HMDB0000208 | 51 | 30915 | C00026 | 5218 |
| **P1,P5-Di(adenosine-5') pentaphosphate (Ap5A)** | C20H29N10O22P5 | 916.0146 | 9.18 | NA | 24893808 | NA | NA | NA |
| **Phosphoenol-pyruvate (PEP)** | C3H5O6P | 167.9824 | 10.52 | HMDB0000263 | 1005 | 44897 | C00074 | 5264 |
| **Phosphohydroxypyruvate** | C3H5O7P | 183.9773 | 10.07 | HMDB0001024 | 105 | 30933 | C03232 | 484 |
| **PRPP** | C5H13O14P3 | 389.9518 | 11.85 | HMDB0000280 | 7339 | 17111 | C00119 | 5274 |
| **Pyruvate** | C3H4O3 | 88.016 | 1.87 | HMDB0000243 | 1060 | 32816 | C00022 | 117 |
| **S-Adenosyl-L-homocysteine (SAH)** | C14H20N6O5S | 384.1216 | 5.43 | HMDB0000939 | 439155 | 16680 | C00021 | 296 |
| **Sarcosine** | C3H7NO2 | 89.0477 | 4.91 | HMDB0000271 | 1088 | 15611 | C00213 | 51 |
| **Scyllo-inositol** | C6H12O6 | 180.0634 | 5.26 | HMDB0006088 | 892 | 10642 | C06153 | NA |
| **ß-Alanine** | C3H7NO2 | 89.0477 | 7.7 | HMDB0000056 | 239 | 16958 | C00099 | 5119 |
| **Succinate** | C4H6O4 | 118.0266 | 8.27 | HMDB0000254 | 1110 | 15741 | C00042 | 114 |
| **Succinyl-CoA** | C25H40N7O19P3S | 867.1313 | 8.58 | HMDB0001022 | 439161 | 15380 | C00091 | 5951 |
| **Taurine** | C2H7NO3S | 125.0147 | 4.44 | HMDB0000251 | 1123 | 15891 | C00245 | 31 |
| **Threonate** | C4H8O5 | 136.0372 | 4.06 | HMDB0000943 | 151152 | 49059 | C01620 | 5891 |
| **Thymidine 5'-monophosphate (TMP)** | C10H15N2O8P | 322.0566 | 7.75 | HMDB0001227 | 9700 | 17013 | C00364 | 6092 |
| **UDP-glucose** | C15H24N2O17P2 | 566.055 | 7.5 | HMDB0000286 | 8629 | 46229 | C00029 | 5278 |
| **UDP-N-acetyl-galactosamine (UDP-GalNAc)** | C17H27N3O17P2 | 607.0816 | 7.73 | HMDB0000304 | 1167 | NA | C00203 | 5293 |
| **UDP-N-acetyl-glucosamine (UDP-GlcNAc)** | C17H27N3O17P2 | 607.0816 | 7.63 | HMDB0000290 | 445675 | 16264 | C00043 | 5281 |
| **Uridine** | C9H12N2O6 | 244.0695 | 1.63 | HMDB0000296 | 6029 | 16704 | C00299 | 90 |
| **Uridine 5'-diphosphate (UDP)** | C9H14N2O12P2 | 404.0022 | 8.88 | HMDB0000295 | 6031 | 17659 | C00015 | NA |
| **Uridine 5-monophosphate (UMP)** | C9H13N2O9P | 324.0359 | 8.4 | HMDB0000288 | 6030 | 16695 | C00105 | NA |
| **Uridine 5'-triphosphate (UTP)** | C9H15N2O15P3 | 483.9685 | 9.26 | HMDB0000285 | 6133 | 15713 | C00075 | 5277 |
| **α-Amino-butyrate (AABA)** | C4H9NO2 | 103.0633 | 4.06 | HMDB0000650 | 439691 | 28797 | C02261 | NA |
| **β-Amino-butyrate (BABA)** | C4H9NO2 | 103.0633 | 5.34 | HMDB0002166 | 439434 | 33094 | C03284 | 6520 |
| **γ-Amino-butyrate (GABA)** | C4H9NO2 | 103.0633 | 7.26 | HMDB0000112 | 223130 | 16865 | C00334 | NA |

**Supplementary Table 1:** Compiled Personal Compound Database and Library (PCDL) for negative ESI mode consisting of 146 metabolites related to glucose intermediary metabolism. Human Metabolome Database (HMDB), Chemical Entities of Biological Interest (ChEBI), Kyoto Encyclopedia of Genes and Genomes (KEGG), NA – not annotated.

| **Primer set** | **Forward (5’-3’)** | **Reverse (5’-3’)** |
| --- | --- | --- |
| β-actin (mouse/rat) | CTGTCGAGTCGCGTCCACCC | ATGCCGGAGCCGTTGTCGAC |
| G6pd (mouse) | GATGCCTTCCACCAAGCTGA | TAGAAGGCCATCCCGGAACA |
| Taldo1 (mouse) | ATCATCAACCTGGGAGGGGA | GTCATGTTGCAGTGGATGCC |
| Pgd (mouse) | CTCCTCGACTCTGCTTCGTC | GCACAGACCACAAATCCATGA |
| Tkt (mouse) | ACGACATCCGAAAGCAGAGT | CATAGGAATTACGGGCAAAGA |
| Glut1 (mouse | ATGGATCCCAGCAGCAAG | CCAGTGTTATAGCCGAACTGC |
| Glut4 (mouse) | GACGGACACTCCATCTGTTG | GCCACGATGGAGACATAGC |
| Gclc (mouse) | GTTATGGCTTTGAGTGCTGCAT | ATCACTCCCCAGCGACAATC |
| Gclm (mouse) | AGTTGACATGGCATGCTCCG | CCATCTTCAATCGGAGGCGA |
| Gsr (mouse) | TGGTAGGAAGCCCACCACAA | ATTTGGGTCCCGTCCAATG |
| Hmox1 (mouse) | CAGCCCCACCAAGTTCAAA | TCAGGTGTCATCTCCAGAGTG |
| Nqo1 (mouse) | GCCCGCATGCAGATCCT | GGTCTCCTCCCAGACGGTTT |
| Txnrd1 (mouse) | GATGCACCAGGCAGCTTTG | TCTTCGACTTTCCAGCCATAGT |
| Cat (mouse) | GCTGAGAAGCCTAAGAACGCAAT | CCCTTCGCAGCCATGTG |
| Gstα2 (mouse) | GCTTGATGCCAGCCTTCTG | GGCTGCTGATTCTGCTCTTGA |
| Nppa (mouse) | CGTGCCCCGACCCACGCCAGCATGGGCTCC | GGCTCCGAGGGCCAGCGAGCAGAGCCCTCA |
| Nppb (mouse) | CCTTTATCTGTCACCGCTGG | CTGTCTCTGGGCCATTTCCT |
| Gclc (rat) | AGTAGAGTTCCGACCAATGGAGG | TATGAGAGGATCACCCTGGTCA |
| Gstα2 (rat) | AAGCTAAAGAAAGACGGGAATTTGAT | GTAGTTGAGAATGGCTCTGGTCTG |
| Hmox1 (rat) | CGACAGCATGTCCCAGCATT | TCTGAAAGTTCCTCATGAACTCAGA |
| Nqo1 (rat) | AGCATTTCAGGGTCGTCCTG | TCTTCTCACCGCCATGGC |
| Txnrd1 (rat) | ACTCAGCAGAGCGGTTCCTC | AAAAGATCGTCACTGCTGATGC |
| Cat (rat) | AGTACAACTCCCAGAAGCCTAAGAAT | CCGTGCTTTACAGGTTAGCTTTTC |
| Pgd (rat) | AGCGCTCAGTCTTTTCGTGT | CAGTGCAATGTCAGCTTGGG |
| G6pd (rat) | GCGGCAACTAAATTCAGAAAA | TCAGGATCCCACACACCTG |
| Taldo1 (rat) | ACCTGGGAGGGAATCCAG | CAGTGTCATGTTGCAGTGGA |
| Tkt (rat) | AACGCTTTCCGTTCCTCTC | TGGCTTATGGTAACCCTCCA |
| Nrf2 (rat) | CTACTCCCAGGTTGCCCAC | CGACTCATGGTCATCTACAAATGG |

**Supplementary Table 2:** List of qPCR primers used in the study.

Original uncropped western blot images:


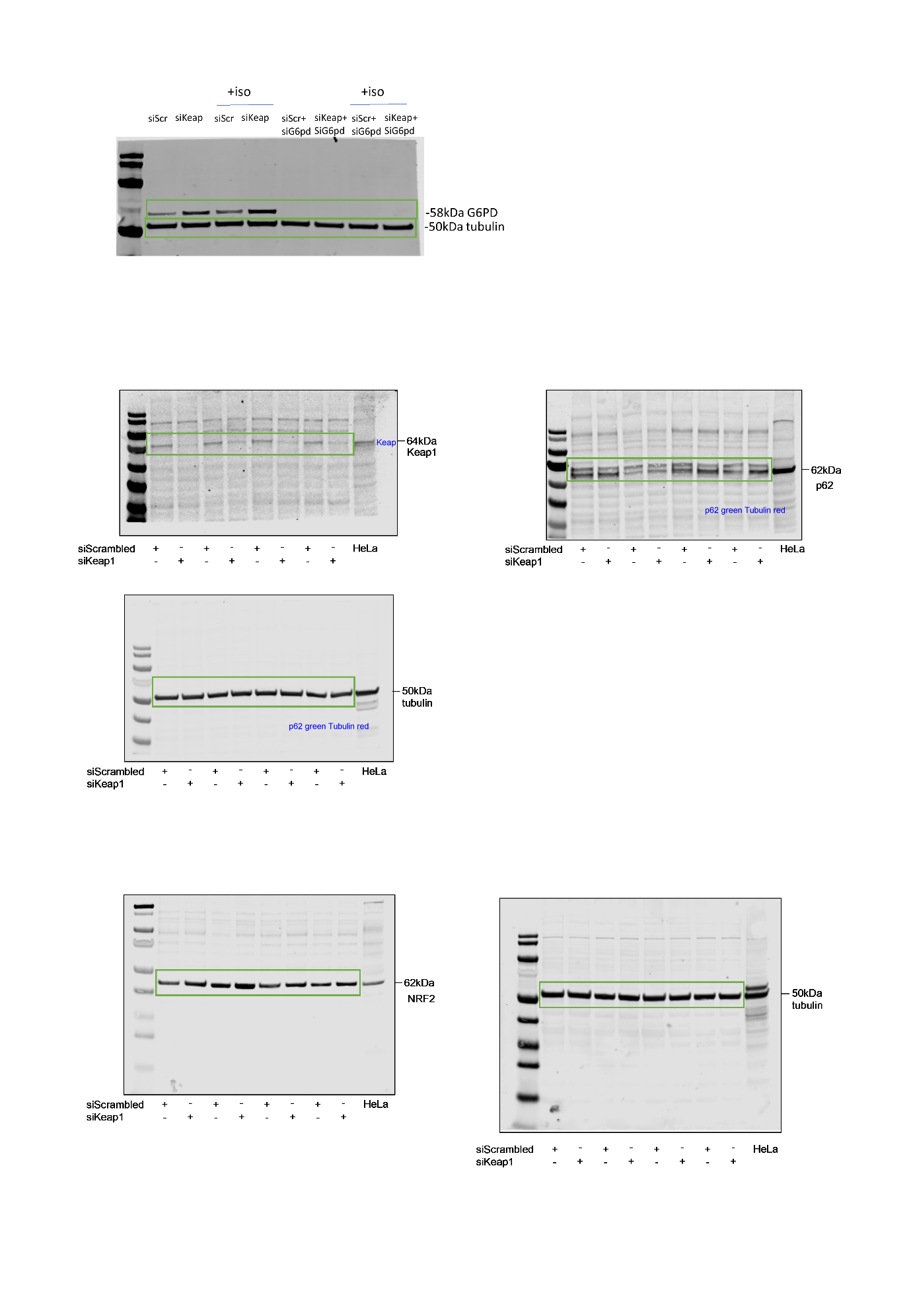


Original uncropped western blot images:


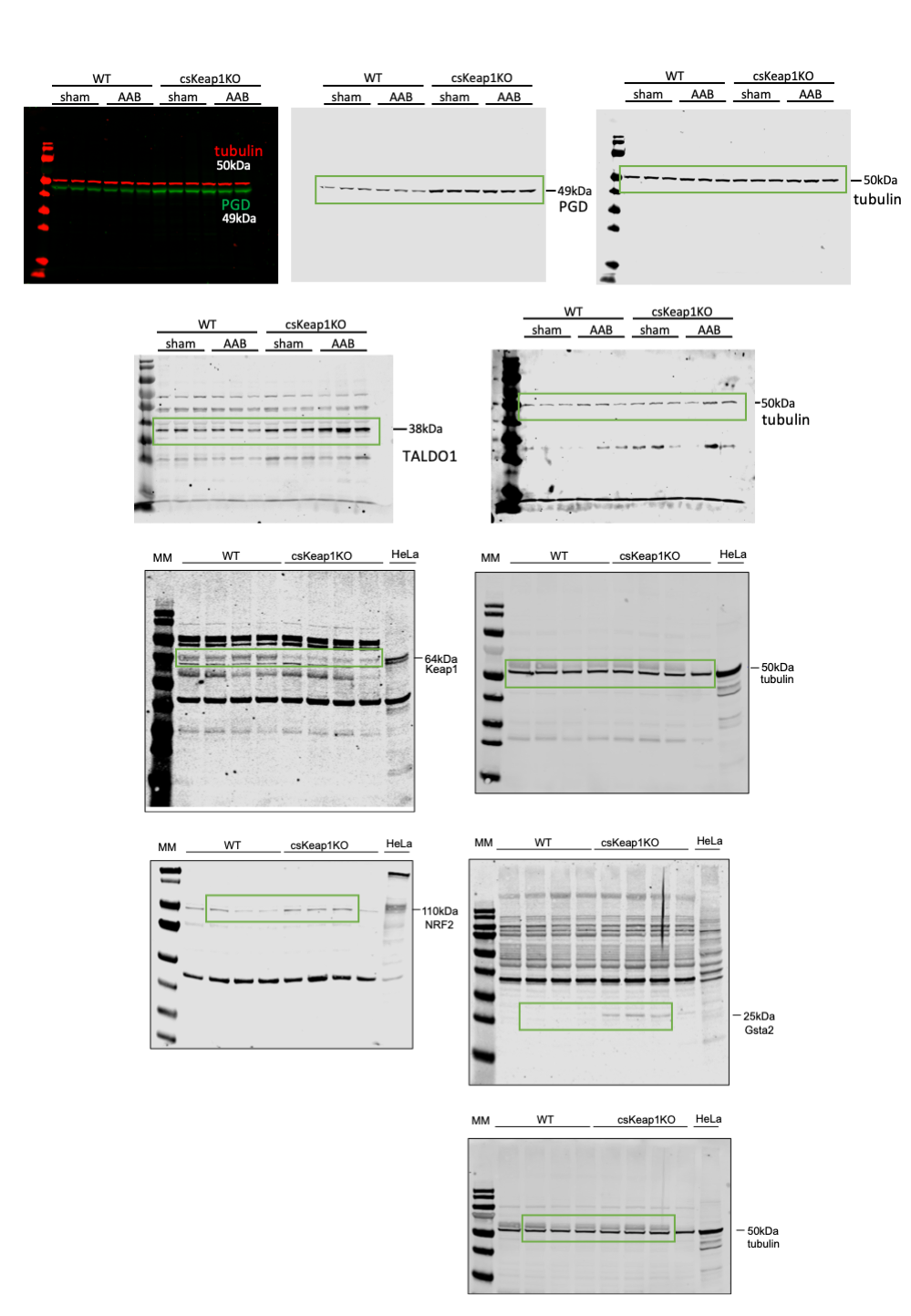

Supplement: cvae250_Supplementary_Data [file cvae250_supplementary_data.docx]
